# Magnetic Cilia with Programmable Beating Patterns for Vortex-Driven Mixing in Microfluidics

**DOI:** 10.1021/acs.langmuir.5c02350

**Published:** 2025-07-31

**Authors:** Dineshkumar Loganathan, Tung OuYang, Chia-Yun Chen, Chia-Yuan Chen

**Affiliations:** † Department of Mechanical Engineering, 34912 National Cheng Kung University, Tainan 701, Taiwan; ‡ Department of Materials Science and Engineering, National Cheng Kung University, Tainan 701, Taiwan

## Abstract

Artificial cilia are widely employed in microfluidic platforms, where their beating motion is harnessed to emulate the fluid transport capabilities of natural motile cilia. In particular, metachronal beating, characterized by phase-shifted motion among adjacent cilia, has proven to be effective for directional fluid transport. However, its potential for micromixing remains limited due to its inherently planar wave propagation, which offers room for improvement in generating strong vortices. To address this, three magnetically actuated artificial cilia carpets are fabricated with identical structural designs featuring spatially varied cilia orientations to embed controlled orientational asymmetry. To realize distinct motion patterns, each carpet is magnetized with a single, unique magnetization profile such that one carpet corresponds to one beating mode, including synchronous, symplectic metachronal, or antiplectic metachronal, and is actuated externally to generate its respective motion. For demonstration purposes, two different experiments are conducted, including micromixing and photocatalytic dye degradation. The results reveal that metachronal motion alone is insufficient to enhance micromixing, thereby highlighting the need for integration with orientational asymmetry. Compared to the aligned cilia carpet (control), superior mixing efficiency of 87% and a 3-fold enhancement in dye degradation are observed in the inclined cilia carpet actuated with antiplectic metachronal motion. This enhanced hydrodynamic activity is further substantiated through μPIV experiments. These findings define metachrony as a dual-function paradigm for both fluid propulsion and vortex-enabled microfluidic mixing.

## Graphical abstract

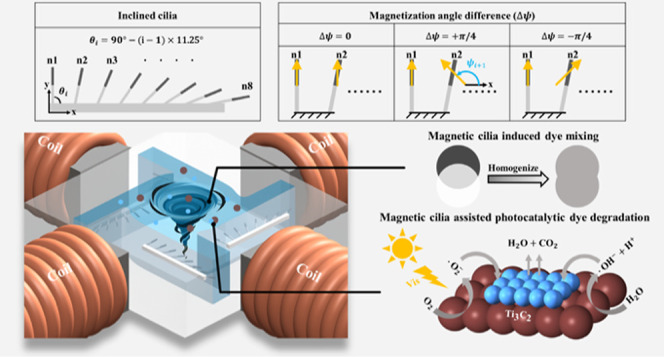

## Introduction

Effective fluid mixing at the microscale remains a fundamental challenge in microfluidic systems due to the predominance of laminar flow regimes and the lack of turbulence at low Reynolds numbers.^1^ In such environments, mixing is primarily driven by molecular diffusion, which is inherently slow and inefficient for many practical applications. To overcome this, a range of micromixer designs has been proposed, broadly classified into passive and active strategies.^2^ Passive micromixers rely on geometric features such as ridges,^3^ herringbone structures,^4^ or serpentine channels^5^ to enhance mixing by stretching and folding fluid streams. However, these devices lack tunability and often require long channel lengths to achieve an acceptable performance. In contrast, active micromixers have been developed to perturb fluid flow and improve mixing efficiency dynamically.^6^ Among active mixing strategies, some approaches utilize internally driven active fluids^7,8^ such as suspensions of motile particles or motor filament systems, which generate spontaneous flows through intrinsic energy conversion mechanisms. In contrast, externally actuated systems like artificial cilia^9,10^ have been developed to facilitate controlled flow manipulation strategies for applications in biomedical,^11–14^ lab-on-a-chip,^15–18^ and microfluidic devices.^19–22^ Moreover, artificial cilia offer a compact, reconfigurable, and controllable alternative to conventional micromixers, enabling rapid and efficient mixing within confined microfluidic environments.

Various actuation techniques have been explored to drive artificial cilia, including magnetic,^23,24^ electric,^25^ pneumatic,^26^ optical,^27^ and acoustic stimulation.^28^ Among these, magnetic actuation has gained prominence due to its remote controllability, ease of implementation, and capability to generate nonuniform deformations in flexible materials. In addition, magnetic cilia have demonstrated superior performance in manipulating fluid flow,^29,30^ particularly by generating larger enclosed trajectories during their motion cycles. This enhanced movement stems from the ability to program the internal response of the material along its length, allowing for significant changes in curvature that closely resemble the motion of biological cilia. Further, magnetically actuated cilia exhibit an ideal balance between structural flexibility and responsiveness to external forces, enabling them to interact effectively with the surrounding fluid.^9^ This property is crucial in promoting coordinated motion patterns across ciliary arrays, ensuring efficient wave propagation and fluid interaction. These highlighted advantages firmly establish the magnetic actuation technique as a promising approach for controlling artificial cilia, especially in replicating their beating patterns to mimic natural cilia, thereby unlocking their full potential for advancing microfluidic research and applications.

Recent studies on magnetically actuated artificial cilia have demonstrated a variety of beating strategies for enhancing mixing in microfluidic systems.^31–38^ One commonly explored approach involves simple oscillatory beating in which artificial cilia undergo a scallop-like back-and-forth motion under a rotating magnetic field. For instance, polydimethylsiloxane (PDMS)-based cilia embedded with carbonyl iron particles were actuated by rotating magnets, generating localized flow disturbances and promoting internal circulation.^35^ In another study, artificial cilia composed of ferromagnetic cobalt particles in an elastomeric matrix were fabricated through a bottom-up method and were shown to bend reversibly in multiple directions under magnetic fields, achieving efficient mixing of viscous fluids.^38^ A distinct mixing strategy involved conical tip trajectories, where cilia swept through tilted circular paths when actuated by rotating fields. This approach was demonstrated by employing PDMS cilia embedded with maghemite nanoparticles and arranged in arrays within a microchannel.^39^ Upon actuation, the conical beating motion of the cilia was reported to generate localized vortices near their bases and induce net fluid flow above the tips, thereby enhancing mixing through advection-driven transport. Beyond this individual cilium beating strategy, coordinated metachronal beating, featuring phase shifts between adjacent cilia, was numerically shown to enhance mixing by generating stronger vortical structures and greater fluid deformation, particularly in the antiplectic mode.^36^ In addition, more advanced implementations involved programmable metachrony, where magnetic cilia were actuated to exhibit laeoplectic and diaplectic waveforms alongside the more commonly studied symplectic and antiplectic modes.^37^ In this numerical study, laeoplectic and diaplectic coordination patterns were found to significantly enhance mixing rates compared to synchronous, antiplectic, and symplectic metachronal waves. Collectively, these beating strategies have demonstrated promising capabilities for manipulating microscale fluid flows by introducing asymmetries during actuation. However, such asymmetries remain effective only within the temporal window of external excitation, thereby limiting their influence outside the actuation period. In contrast, the introduction of a structural or geometric asymmetry, such as orientational variation in cilia alignment, can offer a persistent, actuation-independent strategy which, when combined with the aforementioned excitation-dependent asymmetries, may lead to distinct fluid manipulation behaviors capable of enhancing mixing potential. As such, this approach warrants detailed investigation under the broader scope of alternative strategies for achieving an on-demand mixing capability in microfluidic applications.

In this work, a programmable artificial cilia system was developed by integrating magnetically actuated beating patterns with tailored array configurations to enhance the micromixing performance. In particular, two distinct configurations of magnetically actuated cilia carpets were implemented, each programmed with three different activation modes to generate metachronal waves. The artificial cilia were fabricated by employing a magnetic composite material and actuating through controlled external magnetic fields. In the first design, the cilia were aligned straight in an array without any angular offset. In the second design, each successive cilium was tilted at an incremental angle of 11.25°, resulting in the first cilium standing at 90° and the last cilium positioned at 11.25° relative to the surface. This spatial arrangement introduced an orientational asymmetry (in addition to the metachronal asymmetry), which was observed to influence the flow characteristics. To realize metachronal waves, the cilia beating sequence was programmed with a phase difference between adjacent cilia achieved during the magnetization process. The effect of distinct carpet designs of cilia, coupled with programmed phase-shifted activation, was systematically examined to determine the ideal configuration for achieving dynamic metachronal waves that integrate both fluid transport and mixing. For the purpose of demonstration, two different experiments, including fluid mixing and photodegradation, were conducted and the most effective artificial cilia configuration for the targeted microfluidic applications was identified. The findings from this study contribute to the development of advanced biomimetic cilia systems with enhanced capabilities for microfluidic transport and mixing. These findings hold the potential for applications requiring precise microscale fluid control, including drug delivery, diagnostic devices, and bioengineering solutions.

## Materials and Methods

### The Materials and Fabrication of Magnetic Cilia

Each magnetic cilium was fabricated by using a composite structure consisting of distinct magnetic and nonmagnetic segments. The magnetic portions were composed of a polymer matrix embedded with neodymium–iron–boron (NdFeB) microparticles, while the nonmagnetic regions were formed using pure PDMS. PDMS was selected as the base material due to its favorable characteristics, including biocompatibility, flexibility, optical transparency, elasticity, ease of molding, and cost-efficiency attributes that have made it a widely adopted material in microfluidic applications.^40,41^ Further, each cilium was microfabricated as a slender strip with a square cross-section, measuring 2.8 mm in length, 0.2 mm in width, and 0.2 mm in thickness. The lower 1.4 mm section, measured from the substrate, was fabricated using pure PDMS and served as the nonmagnetic base, whereas the upper 1.4 mm region consisted of a magnetic composite. The magnetic composite was prepared by blending 5 μm NdFeB particles (MQP-15–7, Magnequench International, Inc., Singapore) with PDMS in a weight ratio of 4:1. The PDMS matrix itself was formulated by mixing the elastomer base and curing agent (Sylgard 184, Dow Corning Corp., Midland, MI, USA) in a 10:1 ratio by weight. The fabrication process of the proposed artificial cilia involved four sequential steps, as outlined in Figure 1.

**Figure 1:**
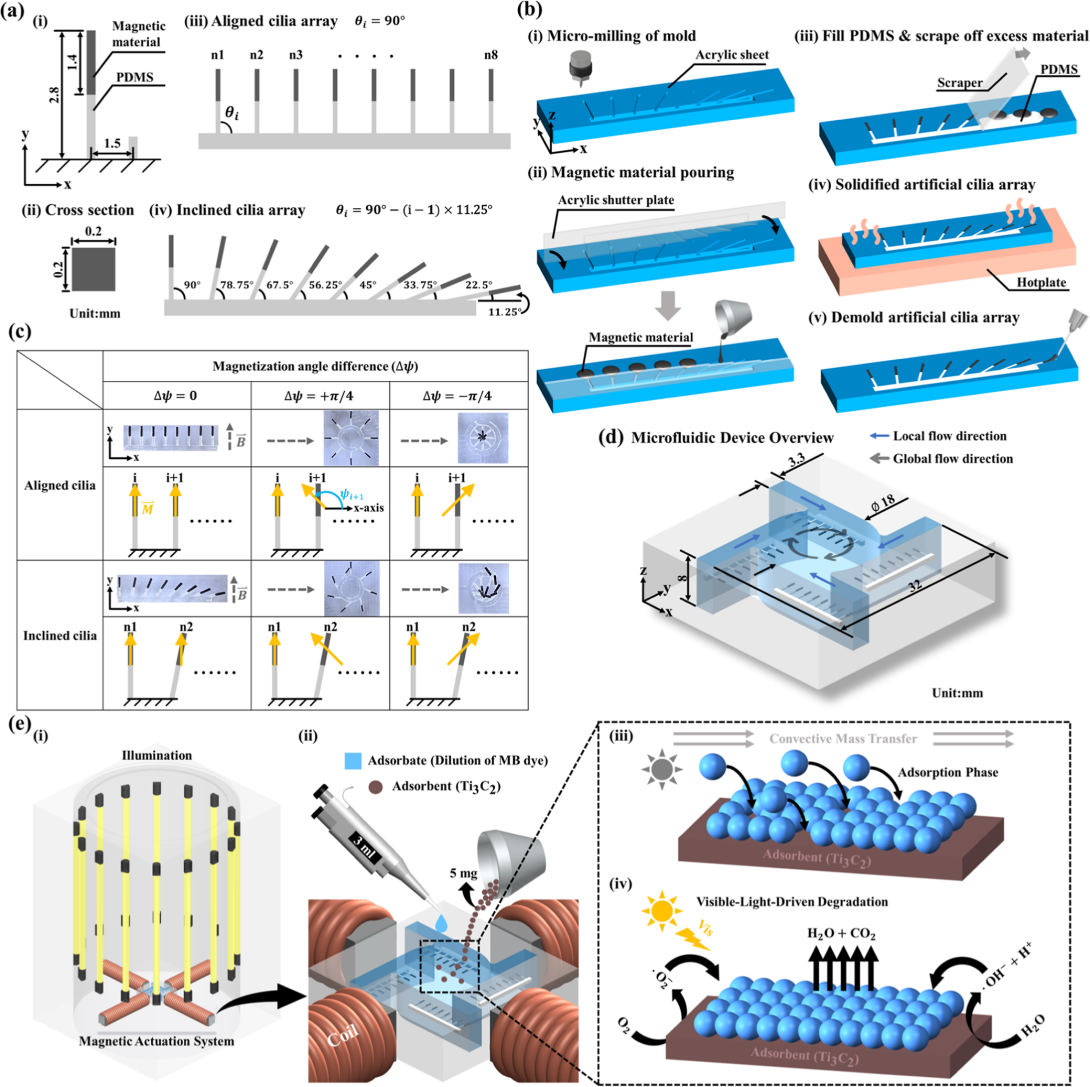
Design, fabrication, actuation modes, and applications of magnetic cilia arrays for microfluidic flow manipulation. (a) Schematic illustrations of individual magnetic cilia and the fabricated two distinct cilia carpet configurations. (i,ii) Geometric features of a single cilium composed of magnetic (NdFeB-PDMS) and nonmagnetic (PDMS) segments, and (iii,iv) Design I with uniformly vertical cilia and Design II with inclined cilia forming a gradient of inclination angles from 90° to 11.25°. (b) Step-by-step fabrication process of artificial cilia arrays featuring a controlled 1:1 length ratio between magnetic and nonmagnetic (PDMS) segments. (i) CNC micromilling of the mold cavity from an acrylic sheet. (ii) Precise masking of the PDMS segment using an acrylic shutter plate, followed by pouring the magnetic composite into the exposed cavity and partial curing. (iii) Removing the shutter plate and subsequent filling of the remaining cavity with PDMS to form the nonmagnetic segment. (iv) Thermal curing of the complete cilia structure on a hot plate. (v) Final demolding of the bilayer artificial cilia array from the mold. (c) Three distinct actuation modes of two different designs of magnetic cilia array that were encoded during the magnetization process. Specifically, mode Δψ = 0 represents the synchronized beating pattern that was preprogrammed by employing a planar magnetization template. Modes Δψ = +π/4 and Δψ = −π/4 represent the antiplectic and symplectic metachronal beating patterns, respectively, that were encoded by employing a circular template. (d) Schematic of the experimental setup that was employed for the fluid mixing embedded with magnetic cilia carpets. (e) Schematic of the photocatalytic degradation experiment by employing Ti_3_C_2_ as an adsorbent under cyclic illumination.

### The Electromagnetic Actuation System

The motion of each magnetic cilium within the array was externally actuated by using a custom-designed electromagnetic control system. This system consisted of four electromagnetic coils symmetrically arranged at 90° intervals around the actuation platform to produce a spatially rotating magnetic field. Each coil was fabricated by winding 1200 turns of copper wire around a carbon steel core with dimensions of 15 mm in length and 2 mm in diameter. The electromagnetic field was generated and regulated through a coordinated setup involving a data acquisition unit (NI DAQ-9174, National Instruments, Austin, TX), an external DC power supply (GPR-3510HD, Instek, Taiwan), and a LabVIEW-based graphical interface developed for precise magnetic control. The data acquisition module facilitated real-time control of the coil current, thereby modulating the magnetic field to achieve the desired ciliary beating patterns. The resulting motion of the artificial cilia was recorded using a charge-coupled device camera (WAT-902H ULTIMATE, WATEC, Japan) fitted with a high-resolution microlens (AF Micro-NIKKOR 60 mm f/2.8D, Nikon, Japan), enabling accurate visualization and documentation of the cilia dynamics during operation.

### The Microparticle Image Velocimetry Experiment

To characterize the fluid dynamic behavior within the magnetic cilia-integrated microfluidic platform, key parameters such as shear stress and circulation were evaluated through the microparticle image velocimetry (μPIV) experiment.^42–44^ This analysis aimed to quantify the flow fields generated by the actuation of the artificial cilia. In this study, fluorescent polystyrene tracer particles with a diameter of 8 μm (Microgenics, Inc., Fremont, CA, USA) were uniformly suspended in deionized water and introduced into the microchannel via a precision-controlled syringe pump. Upon achieving a steady flow condition, the magnetic cilia were actuated to induce fluid motion. The resulting particle displacements were captured by using a high-speed camera (NR4-S2, IDT, Tallahassee, FL, USA) mounted on a fluorescence microscope (BX60, Olympus Corp., Japan). Time-resolved image sequences were subsequently processed using Dynamic Studio 2015 (Dantec Dynamics, Skovlunde, Denmark), a commercial software platform designed for flow visualization and quantitative μPIV analysis. Image preprocessing steps, such as noise suppression and pixel intensity normalization, were carried out to enhance the data quality. The velocity fields were extracted by employing a multipass adaptive cross-correlation algorithm. Specifically, a first-pass interrogation window size of 32 × 32 pixels with 50% overlap was applied for two iterations, followed by a second pass of 16 × 16 pixels with 50% overlap, also for two iterations. This hierarchical processing approach enabled progressive refinement of the velocity field, capturing fine-scale flow features induced by artificial cilia motion. To remove outlier vectors, mean filter-based validation was applied, wherein vectors exceeding 2 times the local root-mean-square (RMS) were excluded and reinserted if within 3 RMS of their neighboring values. Further, this analysis was conducted on 300 image pairs acquired under steady-state actuation conditions, which collectively spanned 18 complete beating cycles of artificial cilia in the array. The final velocity fields were then ensemble-averaged to suppress noise and capture representative cycle-averaged flow behavior associated with each cilia configuration.

### The Micromixing Experiment

The micromixing behavior of the system was evaluated by introducing methylene blue (MB) dye into a mixing chamber partially prefilled with distilled water. The chamber was configured with four magnetically actuated cilia carpets arranged symmetrically along its lateral edges. Each carpet was affixed to the bottom surface of the chamber at an approximate depth of 4 mm from the fluid–air interface, with the working fluid comprising a 40% aqueous glycerol solution. A micropipette was employed to inject 10 μL of concentrated MB dye at a targeted location within the chamber. Following dye injection, the magnetic cilia arrays were actuated by employing the previously described custom electromagnetic coil system, initiating the mixing process. The resulting dynamics were recorded by using a high-resolution camera. To quantify the mixing performance, the captured image sequences were analyzed using ImageJ software (National Institutes of Health, USA). Each image was then cropped to isolate the region of interest corresponding to the microchannel mixing zone. By employing the built-in Histogram function in ImageJ, pixel intensity statistics, including the pixel count (the total number of pixels within the selected ROI (*n*)), mean (the average gray value across all pixels (*m̅*)), and standard deviation (representing the dispersion of pixel intensities from the mean (σ)) of the grayscale images within the ROI, were extracted. These extracted values were directly substituted into eq 1 to compute the mixing efficiency.

1 $$mixing\;efficiency\;\left(\%\right)=\left(1-\frac{1}{\mathrm{m̅}}\sqrt{\frac{\sum_{i}^{n}{\left(m_{i}-\mathrm{m̅}\right)}^{2}}{n}}\right)\times 100\%$$

2 $$\left(\sigma \right)=\sqrt{\frac{\sum_{i}^{n}{\left(m_{i}-\mathrm{m̅}\right)}^{2}}{n}}$$

Note that the standard deviation can be written as eq 2.

Upon substituting ([Disp-formula eq2]) in ([Disp-formula eq1])­

3 $$mixing\;efficiency\;\left(\%\right)=\left(1-\frac{\sigma }{\mathrm{m̅}}\right)\times 100\%$$

A mixing efficiency of 100% indicates complete homogeneity (i.e., uniform dye distribution), while 0% corresponds to a completely unmixed state.

### Statistical Analysis

To ensure experimental reproducibility, all measurements, including mixing efficiency and photocatalytic dye degradation, were conducted in triplicate (n = 3 independent trials) for each of the tested artificial cilia configurations. Further, statistical analysis was carried out using SPSS software (SPSS 17.0.0, Chicago, IL, USA) to compare results among different cilia designs and actuation modes. The normality of each data set was assessed using the Shapiro–Wilk test. Subsequently, paired samples *t* tests were conducted to evaluate statistically significant differences between selected configuration pairs. Statistically significant differences were defined as (*p* ≤ 0.01, denoted by **) and (0.01 < *p* ≤ 0.05, denoted by *).

## Results and Discussion

### The Design and Actuation Modes of Magnetic Cilia

To develop an improved solution for fluid manipulation strategies in microfluidic environments, particularly for fluid mixing, two distinct configurations of magnetic cilia arrays were fabricated, and the dynamics of their beating motions were investigated in this study. Each array was constructed by assembling eight individual magnetic cilia, where each cilium was designed as a slender microstrip with a square cross-section, measuring 2.8 mm in length, 0.2 mm in width, and 0.2 mm in thickness, as illustrated in Figure 1a (i,ii). Further, each cilium comprised both magnetic and nonmagnetic segments. The magnetic regions were composed of a composite material incorporating neodymium–iron-boron (NdFeB) particles embedded in PDMS, whereas the nonmagnetic sections were formed using pure PDMS. Within the 2.8 mm length of each cilium, the lower 1.4 mm segment (measured from the substrate) remained nonmagnetic, while the upper 1.4 mm section constituted the magnetic region. As depicted in Figure 1a (iii,iv), the two fabricated array designs were referred to as aligned cilia and inclined cilia configurations. In aligned cilia, all eight cilia were vertically affixed to the substrate along their longitudinal axis, maintaining an interciliary separation distance of 1.5 mm. Consequently, each cilium formed a 90° angle (θ*i*) with the array surface. In contrast, in inclined cilia, the cilia were positioned at progressively decreasing inclination angles. Specifically, the first cilium (*n* = 1) was oriented at 90°, while the inclination angle of each subsequent cilium was reduced by 11.25°, resulting in the eighth cilium (*n* = 8) being positioned at 11.25°. The rationale behind these configurations was to evaluate and compare the flow manipulation characteristics associated with each array design. Since all cilia in aligned cilia were positioned perpendicular to the substrate, no orientational asymmetry was introduced. Instead, only metachronal asymmetry can be induced (based on the actuation mode, which is discussed in detail in subsequent sections). Conversely, in inclined cilia, orientational asymmetry was introduced by affixing each cilium to the substrate at varying, nonuniform inclination angles, in addition to the induction of metachronal asymmetry. This variation in the design configurations was expected to enhance the flow manipulation capabilities, thereby improving the fluid mixing efficiency. Meanwhile, to fabricate these cilia arrays, a micromilling process (Figure 1bi) was employed as a first step to create the mold cavity with the desired geometric configuration. Following mold preparation, PDMS and a mixture of PDMS-NdFeB composites were synthesized and thoroughly homogenized to achieve a uniform dispersion of magnetic constituents. These formulations were then systematically introduced into the mold cavities (in order to achieve the intended length ratio between magnetic and nonmagnetic sections of cilia, an acrylic shutter plate was employed to mask the PDMS segment, followed by pouring the magnetic composite into the exposed cavity and partial curing (Figure 1bii) to ensure complete infiltration, while excess material was carefully removed by employing a precision scraping technique (Figure 1biii) to maintain dimensional integrity. Subsequently, the mold was placed on a heated plate and subjected to controlled thermal curing at a temperature of 85 °C for 2 h to facilitate polymer cross-linking and material solidification (Figure 1biv). Once the curing process was completed, the mold was gradually cooled to room temperature to prevent thermal stress-induced deformations. Finally, the cured cilia array was carefully removed from the mold (Figure 1bv), ensuring structural integrity without compromising the flexibility or magnetic responsiveness of the fabricated cilia. To achieve controlled ciliary motion, three distinct magnetization modes, designated mode Δψ = 0, mode Δψ = +π/4, and mode Δψ = −π/4, were implemented, as conceptually illustrated in Figures 1c and S1 and Section S1. A permanent magnet positioned proximal to the cilia array was employed to magnetize the cilia, thereby enabling the implementation of the designated actuation modes (The corresponding directions of the applied magnetic field are indicated schematically by dashed arrows annotated with a ‘B’ field symbol adjacent to each template). In mode Δψ = 0, a standard magnetization protocol was utilized. During this process, the fabricated cilia array was affixed to a planar magnetization template, as shown in Figure 1c (mode Δψ = 0). Given the vertical alignment of all cilia along their longitudinal axes and the consistent orientation maintained throughout the magnetization process, uniform magnetization vectors were induced across the entire array (i.e., the angular difference in magnetization between adjacent cilia was zero Δφ = 0). Consequently, synchronized cilia motion was elicited in mode Δψ = 0 (a detailed discussion is provided in the subsequent sections). Conversely, modes Δψ = +π/4 and Δψ = −π/4 were preprogrammed to induce phase-lagged ciliary motion with opposing wave propagation directions. Similar to mode Δψ = 0, the beating patterns of modes Δψ = +π/4 and Δψ = −π/4 were also encoded during the magnetization procedure. However, in contrast to mode I, a circular magnetization template was employed to induce the specific magnetization profiles necessary for achieving modes Δψ = +π/4 and Δψ = −π/4, as illustrated in Figures 1c and S2. In particular, during the magnetization process of mode Δψ = +π/4, the cilia array was conformed to the outer surface of the circular template, whereas for Δψ = −π/4, it was conformed to the inner surface of the circular template. Although the magnetization angles imposed on each cilium were identical (0°, 45°, and 90°), the resulting magnetization vector orientations were rendered opposite between modes Δψ = +π/4 and Δψ = −π/4. For instance, the directions of the magnetization vectors (Δφ) in Δψ = +π/4 and Δψ = −π/4 were configured to be opposite with Δφ assigned values of +π/4 and −π/4, respectively. Consequently, when the magnetized cilia arrays from Δψ = +π/4 and Δψ = −π/4 were subjected to an external magnetic field, distinct metachronal wave propagation directions were observed. Specifically, in mode Δψ = +π/4, an antiplectic metachronal wave was generated, where the wave propagation direction was the same as the direction of the elastic (recovery stroke) of artificial cilia (Figure S3). Conversely, in mode Δψ = −π/4, a symplectic metachronal wave was produced, wherein the wave propagation direction was opposite to the direction of the elastic (recovery stroke) of artificial cilia (Figure S3). Meanwhile, for the purpose of demonstration, two experimental applications, namely, fluid mixing and photocatalytic degradation, were conducted, as depicted in Figure 1d,e. A comprehensive analysis of these experimental observations and their implications for microfluidic applications is presented in the subsequent sections.

### The Characterization of Artificial Cilia Motion Dynamics across Distinct Actuation Modes

The functional adaptability of artificial cilia can be substantially enhanced through the application of discrete actuation modes, each designed to generate specific fluidic responses within microscale environments. Prior to analyzing the emergent behaviors of a complete cilia carpet, it is essential to characterize the fundamental motion dynamics of a single cilium. For this purpose, the aligned cilia carpet configuration was selected as a representative design to experimentally capture and illustrate the characteristic motion of an individual cilium. A single cilium exhibits a rhythmic beating pattern, which can be segmented into two distinct phases such as the effective (magnetic) stroke, induced by the external magnetic field, and the recovery (elastic) stroke, characterized by return motion (in the absence of a magnetic field), as illustrated in Figure 2a. In the present study, cilium actuation was achieved by employing a rotating magnetic field generated by a custom-designed electromagnetic system^45^ comprising four electromagnetic coils arranged circumferentially around the experimental platform. This rotating field was generated by precisely regulating the phase differences of the input currents supplied^46^ to the individual coils. Each complete rotation of the magnetic field was observed to induce one full actuation cycle of the cilium, consisting of two distinct strokes (phases) such as (i) a magnetic field-induced torque-generated motion phase of cilium that was oriented along the direction of the supplied magnetic field (Figure 2a (i)) and (ii) a subsequent elastic recovery phase, wherein the cilium was observed to return to its initial position as a result of the release of stored elastic energy (Figure 2a (ii)). These two phases closely resembled the power and recovery strokes characteristic of natural ciliary motion, as mentioned. The underlying mechanism responsible for this behavior in the magnetic ciliary system can be attributed as (i) during the magnetic deflection phase, actuation of the surrounding coil generated a magnetic field that was oriented opposite to the magnetization vector of the cilium, thereby inducing a magnetic torque that drove its deflection toward the direction of the rotating field. (ii) The subsequent recovery phase was driven by elastic restoring forces and was triggered when the cilium reached a geometrically constrained position where alignment with the rotating field was no longer feasible. At this point, a rapid return to the original configuration was induced by the release of stored elastic energy governed by the anchorage conditions and structural compliance of the cilium. Regardless of the specific actuation mechanism employed, the two fundamental strokes have been consistently observed across all artificial cilia types and their corresponding beating patterns.^9,47^ However, the spatiotemporal characteristics, including the duration of each stroke and the interciliary spacing during cyclic motion (both of which are intrinsically linked to ciliary motion dynamics), can vary significantly among the distinct beating patterns. The rest of this section is dedicated to discussing these motion characteristics associated with distinct actuation modes based on experimental observations. To facilitate a systematic comparison, three representative cilia (numbered 3, 4, and 5 out of a total of eight (Figure 2b) were selected from aligned cilia configuration, and two key parameters were evaluated, including (i) the temporal variation in the *x*-position of each cilium tip (*x* ^tip^) during the magnetic and elastic strokes and (ii) the interciliary spacing between adjacent cilia (*d*_*x*_^tip^) during each respective stroke phase. The findings, illustrated in Figure 2b, employed shaded regions to demarcate the magnetic (gray) and elastic strokes (yellow). For mode Δψ = 0, it can be seen from Figure 2b (i, lower panel) that there were no observable differences in the value of *x* ^tip^ among the considered cilia (*n* = 3, 4, and 5). Despite differing durations between the magnetic and elastic strokes, this timing was observed to be consistent across all three cilia. For instance, during the magnetic stroke, the tip of cilium *n* = 3 was displaced 1 mm along the *x*-axis within 0.1 s, while the subsequent elastic stroke made the cilia return to their initial position within 0.06 s. Similar durations for the magnetic and elastic strokes were further observed in cilia 4 and 5, leading to synchronized motion across all of the cilia in the carpet. This synchronous motion was attributed to the magnetization profiles associated with mode Δψ = 0 and the uniform vertical anchoring conditions of each cilium in the carpet. Despite the phase coherence, the motion was observed to remain nonreciprocal, as evidenced by the unequal durations of the magnetic and elastic strokes. This disparity between the durations of the magnetic and elastic strokes gave rise to a characteristic temporal asymmetry, recognized as one of the three asymmetries (spatial, orientational, and temporal) that have been reported to emerge at the single-cilium level.^7^ Collectively, mode Δψ = 0 actuation was defined by synchronized, nonreciprocal motion with temporal asymmetry (Video S1). Apart from these nonreciprocal and asymmetric motions, the ciliary system that is employed for low Reynolds number applications also depends on the third parameter that measures the sustained interactions between moving surfaces and the fluid medium that could directly impact the propulsion efficiency, mixing capability, or particle transport effectiveness. The latter parameter can be quantified by determining the interciliary (between the adjacent cilia) spacing during cyclic motion (denoted as *d*_*x*_^tip^), as illustrated in Figure 2b (i, upper panel). Further, this metric implicitly indicates the dynamic packing density of beating structures within the cilia carpet. Meanwhile, the lower value of *d*_*x*_^tip^ corresponds to a closer spacing between neighboring cilia tips occurring at a specific phase in the beating cycle, which influences the timing and extent of the fluid interaction during actuation. In mode Δψ = 0, the *d*_*x*_^tip^ value was observed to remain constant (1.5 ± 0.15 mm, Figure 2b (i, upper panel)) throughout both the magnetic and elastic stroke phases, indicating a stable packing configuration. This invariance suggested that the cilia carpet maintained a uniform density during actuation, with no significant temporal modulation of the fluid-contact area. As a result, this behavior can support steady-state fluid manipulation capabilities. In contrast to mode Δψ = 0, mode Δψ = +π/4 was observed to exhibit out-of-phase beating among the considered cilia, as shown in Figure 2b (ii, lower panel). Unlike mode Δψ = 0 (where all cilia reached the peak value of *x* ^tip^ simultaneously), mode Δψ = +π/4 was characterized by a sequential tip displacement. Specifically, cilium number 5 was observed to reach its peak at 0.05 s, followed by cilium 4 at 0.08 s and cilium 3 at 0.1 s. This sequential pattern indicated the formation of a metachronal wave propagating from the distal to the proximal end of the cilia carpet (right (*n* = 8) to the left (*n* = 1)), characteristic of an antiplectic motion. Despite the phase differences, temporal asymmetry was still maintained with longer magnetic strokes relative to the elastic strokes. Thus, mode II actuation was defined by antiplectic metachronal motion combined with temporal asymmetry (Video S1). In addition, unlike mode Δψ = 0, the value of *d*_*x*_^tip^ in mode Δψ = +π/4 was observed to decrease during the elastic stroke (from 1.94 ± 0.09 mm in the magnetic stroke to 0.8 mm, Figure 2b (ii, upper panel)), implying an increase in ciliary density during the recovery stroke. Further, similar to mode Δψ = +π/4, mode Δψ = −π/4 was also observed with the out-of-phase beating pattern among the considered cilia, as depicted in Figure 2b (iii, lower panel). However, the sequence of attaining the peak *x* ^tip^ value was reversed. In mode Δψ = −π/4, cilium number 3 reached its peak *x* ^tip^ value at 0.05 s, followed by cilia 4 and 5 at 0.08 and 0.1 s, respectively. This pattern indicated the propagation of the metachronal wave propagating from the proximal to the distal end of the cilia carpet (left (*n* = 1) to the right (*n* = 8)), characteristic of a symplectic motion. Despite the phase differences, temporal asymmetry was still maintained, with longer magnetic strokes relative to the elastic strokes. Thus, mode Δψ = −π/4 actuation was characterized by symplectic metachronal motion combined with temporal asymmetry (Video S1). During mode Δψ = −π/4 actuation, the value of *d*_*x*_^tip^ was observed to increase during the elastic stroke (from 1.07 mm in the magnetic stroke to 2.13 ± 0.06 mm in the elastic stroke, Figure 2b (iii, upper panel)), resulting in reduced cilia density during the recovery phase of cilia. Furthermore, Figure 2c (i–iii) displays representative experimental snapshots captured during the actuation cycle to examine the dynamic variation in interciliary spacing between adjacent cilia (specifically between cilia numbered *n* = 3 and *n* = 4) for all the considered modes, respectively. These images further provide qualitative validation for the trends of intertip distance (*d*_*x*_^tip^) shown in the top panels of Figure 2b (i–iii). Thus, while both symplectic (mode Δψ = −π/4) and antipelctic (mode Δψ = +π/4) metachronal wave propagations have been typically associated with enhanced directional flow,^15^ the decreased contact area during the recovery stroke may limit fluid manipulation benefits in mode Δψ = −π/4 relative to mode Δψ = +π/4. To further validate the programmed control over ciliary motion and quantify the relationship between magnetization profiles and the resulting motion, the phase difference of motion (ΔΦ) was evaluated as a function of the magnetization angle difference (Δψ) imposed during fabrication (Figure 2d). The experimental phase difference was calculated by employing eq 4.^48^

4 $$\Delta \Phi =2\pi \times \left(\frac{\Delta t}{T}\right)$$

where Δ*t* represents the time delay between the peak displacements of adjacent cilia, and *T* is the period of one complete ciliary beat cycle (as defined in Figure 2b, ii). For instance, ΔΦ = 0 denotes synchronized ciliary motion, while nonzero values indicate a time delay between adjacent cilia, giving rise to metachronal waves. A value of ΔΦ = π (180°) signifies a completely out-of-phase beating. As shown in Figure 2d, the observed phase differences (ΔΦ) closely matched the programmed magnetization angle differences (Δψ), though with sign inversions in modes Δψ = +π/4 and Δψ = −π/4. Specifically, Δψ = +π/4 in mode II produced a (ΔΦ) of −π/4, indicating antiplectic wave propagation, whereas Δψ = −π/4 in mode III yielded a (ΔΦ) of +π/4, corresponding to symplectic motion. This sign inversion arose from the chosen convention for defining the phase direction relative to the ciliary power stroke. Importantly, the consistency in magnitude between programmed and observed phase differences confirms the system’s precise control over metachronal beating patterns. These distinct actuation modes form the basis for subsequent performance evaluations in mixing and photodegradation, where the ideal mode was determined by hydrodynamic effects, such as shear stress generation and circulation.

**Figure 2:**
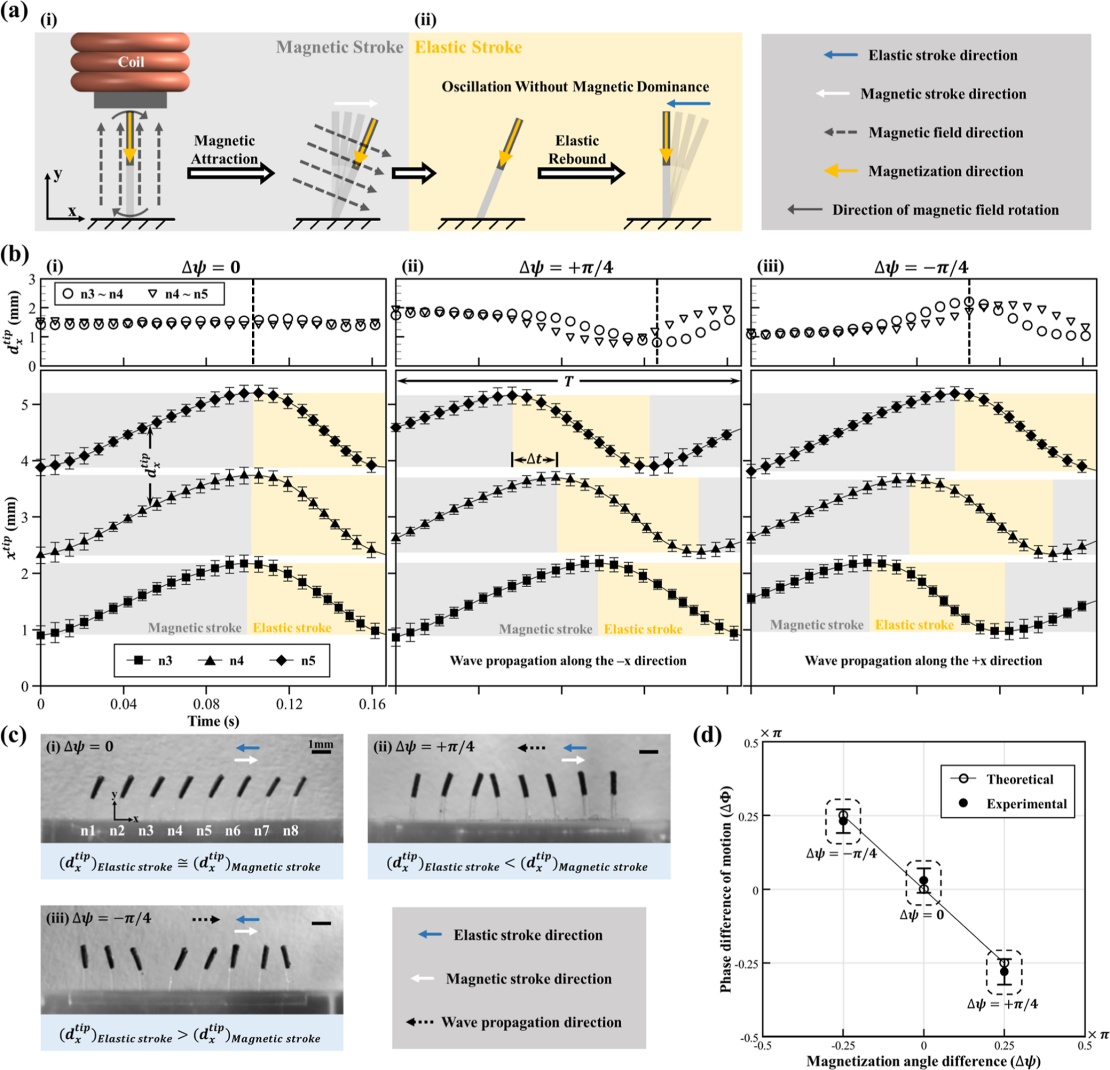
Characterization of single and collective artificial cilia motion dynamics under three distinct actuation modes. (a) Schematic illustration of the fundamental motion of a single artificial cilium under a rotating magnetic field, comprising (i) the magnetic (effective) stroke, where the cilium was deflected by an external magnetic field, and (ii) the elastic (recovery) stroke, where it was passively returned to its initial configuration due to elastic restoring forces. (b) Experimental quantification of spatiotemporal motion characteristics of three representative cilia (*n* = 3, 4, and 5) out of eight in the carpet for all different actuation modes. Note that this analysis was performed by employing the aligned cilia configuration as a representative structure for characterizing fundamental motion dynamics. The top panels show the variation in interciliary spacing between adjacent cilia tips (*d*_*x*_^tip^), while the lower panels present the *x*-position of cilia tips (*x* ^tip^) during one full actuation cycle. Shaded regions denote the magnetic stroke (gray) and elastic stroke (yellow). Mode Δψ = 0 was observed to exhibit synchronized, nonreciprocal beating with a constant value of *d*_*x*_^tip^ (representing a uniform displacement across cilia). In contrast, mode Δψ = +π/4 demonstrates antiplectic metachronal wave propagation (distal-to-proximal) with temporally staggered tip displacement and a decrease in the value of *d*_*x*_^tip^during the elastic stroke. Meanwhile, mode Δψ = −π/4 shows symplectic metachronal motion (proximal-to-distal) with a reversed displacement sequence and an increase in the value of *d*_*x*_^tip^ during the elastic stroke. The error bar represents a unit standard deviation in both directions, calculated from the results of three independent experiments. (c) Representative experimental snapshots of magnetic cilia carpet (aligned cilia configuration) at selected time points during the beating cycle for each mode, which qualitatively validates the observed changes in interciliary spacing (between cilia *n* = 3 and *n* = 4) (*d*_*x*_^tip^) described in panel (b). These images highlight the differences in local cilia density during actuation, with mode Δψ = +π/4 enhancing the fluid-contact area during recovery, while mode Δψ = −π/4 was observed to reduce it. The scale bar represents 1 mm. (d). This figure shows the relationship between the programmed (or magnetized Δφ) and observed phase differences (ΔΦ) for different actuation modes. The error bar represents a unit standard deviation in both directions, calculated from the results of three independent experiments.

### An Assessment of Fluid Mixing Behavior Induced by Aligned and Inclined Cilia Configurations across all Actuation Modes

In microenvironments, where diffusion often limits effective fluid manipulation, the generation of controlled mixing has been regarded as essential for improving fluid handling.^49,50^ Meanwhile, the term mixing is defined as the degree of homogeneity achieved within the fluid volume over time, and it can be quantitatively assessed using a parameter referred to as mixing efficiency.^51–53^ This efficiency was assessed in this study to systematically investigate the flow mixing performance associated with different cilia carpet configurations and actuation patterns. Further, to demonstrate the mixing experiment, MB dye was introduced into the mixing chamber, which had been partially filled with distilled water, as illustrated in the inset of Figure 3a (i). The chamber was configured with four cilia carpets symmetrically positioned along its lateral boundaries, each located at an estimated depth of 4 mm beneath the fluid’s free surface. As previously described, the cilia were actuated by employing a rotating magnetic field generated by four surrounding electromagnetic coils, as also depicted in Figure 3a (i). The evaluation of the mixing efficiency was first carried out by actuating aligned cilia carpets under three distinct actuation modes. The temporal evolution of mixing efficiency under the mentioned conditions is shown in Figure 3a (i). Although an increasing trend was consistently observed across all modes, mode Δψ = 0 demonstrated a markedly higher mixing efficiency (84%) at 60 s compared to mode Δψ = +π/4 (75%) and mode Δψ = −π/4 (71%). The representative snapshots that were captured during the experiment (at 60 s) are presented in Figure 3a (ii), which further supports these findings. To further validate the experimental observations and to elucidate the associated hydrodynamic behavior, an μPIV analysis was conducted. A detailed description of the experimental μPIV setup and methodology is provided in the “Materials and Methods” section. Among the three modes, the configurations yielding the highest and lowest mixing efficiencies, such as mode Δψ = 0 and mode Δψ = −π/4, were selected for the μPIV-based hydrodynamic investigation. In particular, the shear stress distribution, indicative of the mixing performance, was computed as an ensemble average over 18 actuation cycles to ensure statistical consistency and reduce the effects of transient variations (Figure 3b (i,ii)). The maximum shear stress was measured to be 35 mPa in the aligned cilia array actuated under mode (Δψ = 0), whereas a value of 22 mPa was recorded for the inclined cilia array actuated under mode (Δψ = −π/4). This contrast suggests that the synchronous actuation mode was more effective in promoting mixing effects compared with the symplectic configuration. To further understand the hydrodynamic behavior associated with each mode in the aligned cilia array configuration, a separate flow field analysis was conducted by employing a single array of artificial cilia actuated under the three metachronal modes (Section S2 and Figure S4, top panel). This additional evaluation revealed that the synchronous mode (Δψ = 0) produced a compact and intense vortex with maximum vorticity values along with higher spatially averaged velocities compared to the antiplectic and symplectic modes. In contrast, the latter two modes were observed to generate more elongated and less coherent vortices with relatively lower vorticity magnitudes, indicating a weaker rotational strength.

**Figure 3:**
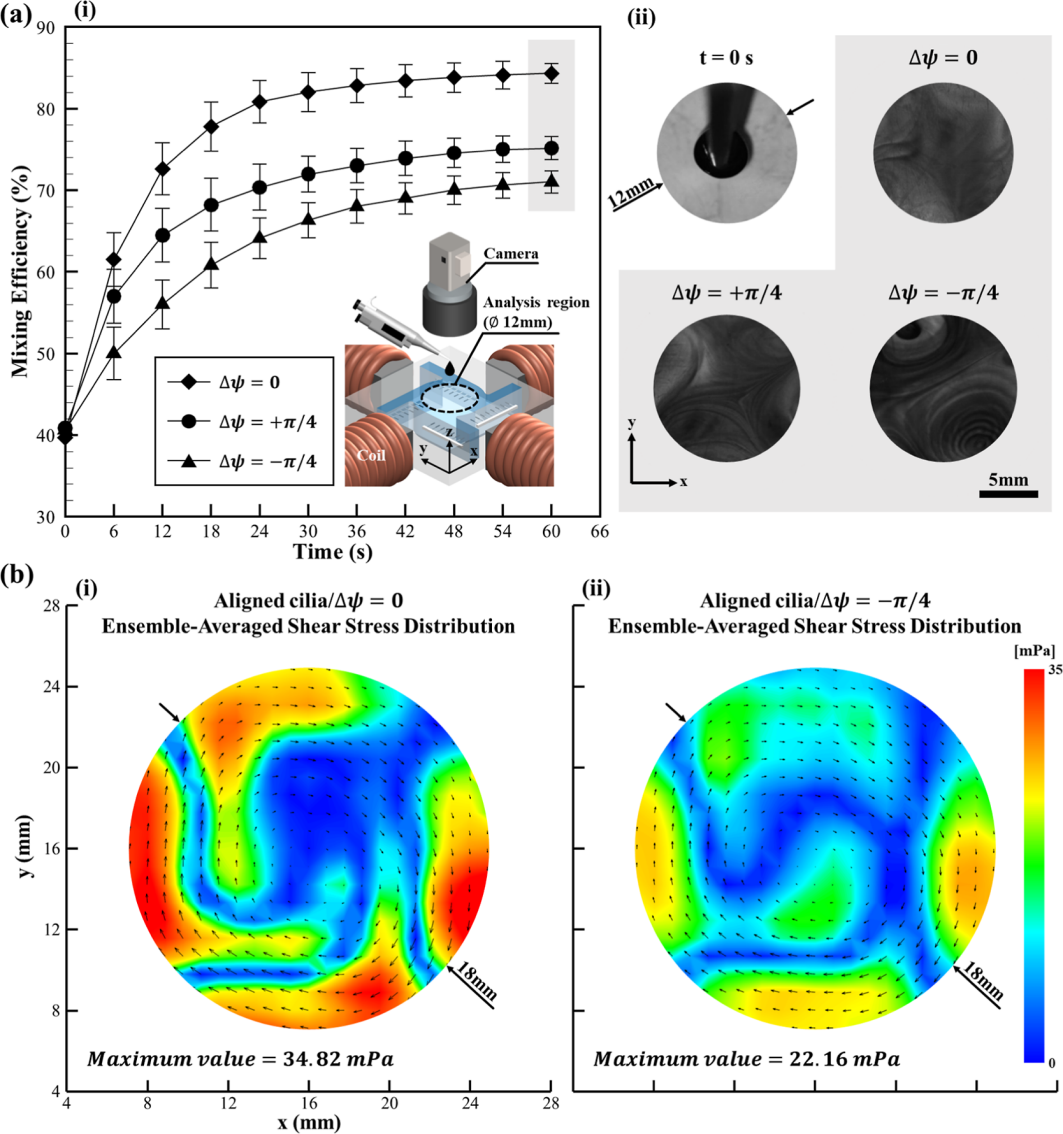
Analysis of mixing performance associated with aligned cilia array configuration and its respective hydrodynamic behavior. (a) A comparison of mixing efficiency associated with aligned cilia under different actuation modes. (i) Illustration of the temporal evolution of mixing efficiency over 60 s for aligned cilia that was actuated under all the modes. Mode Δψ = 0 was observed to reach the highest mixing efficiency (84% at 60 s) compared to mode Δψ = +π/4 (75%) and mode Δψ = −π/4 (71%), suggesting that synchronized motion was more effective for mixing in this configuration. The error bar represents a unit standard deviation in both directions, calculated from the results of three independent experiments. (ii) Representative snapshots of the dye distribution within the mixing chamber at *t* = 0 s (initial condition) and at *t* = 60 s for each actuation mode. The scale bar represents 5 mm. (b) The ensemble-averaged shear stress distribution maps for selected actuation modes. (i) Shear stress distribution map for aligned cilia/Δψ = 0, representing the ensemble average over 18 actuation cycles. The maximum shear stress for mode Δψ = 0 was observed to reach 35 mPa. (ii) The maximum shear stress for mode Δψ = −π/4 was observed to be 22 mPa.

In the “inclined cilia” configuration, since each artificial cilium was embedded into the array with varying inclination angles, a form of orientational asymmetry was established across the array. Specifically, the first cilium (*n* = 1) was positioned perpendicularly (90°), while the inclination angle of each subsequent cilium was reduced by 11.5°, resulting in the eighth cilium (*n* = 8) being oriented at 11.5° relative to the surface. Meanwhile, as previously determined in aligned cilia design, mode Δψ = −π/4 had been found to be ineffective in generating localized shear stress and was therefore excluded from further analysis in inclined cilia. The evolution of mixing performance associated with inclined cilia under both mode Δψ = 0 and mode Δψ = +π/4 actuation conditions was assessed and is presented in Figure 4a. After 60 s of mixing operation, inclined cilia/Δψ = +π/4 was observed to attain the maximum mixing efficiency of 87%, whereas the same design under mode Δψ = 0 was found to be 78%. Notably, the maximum efficiency achieved by aligned cilia/Δψ = 0 was 85% (Figure 3a (i)). Until the 30 s of mixing operation, aligned cilia/Δψ = 0 was observed to remain superior to the inclined cilia configuration; however, beyond this point, inclined cilia/Δψ = +π/4 was observed to exhibit a modest improvement, ultimately surpassing the performance of aligned cilia (Video S1). To further substantiate these quantitative findings, representative experimental snapshots captured at 60 s are provided in the inset of Figure 4a. The dye distribution in inclined cilia/Δψ = +π/4 exhibited a slightly more uniform dispersion and deeper color intensity, consistent with its marginally higher mixing efficiency. In comparison, the images corresponding to aligned cilia/Δψ = 0 and inclined cilia/Δψ = 0 displayed relatively lighter regions and less uniform dye profiles, aligning with the measured efficiency values. To further support this claim, the flow circulation was evaluated, and their results are depicted in Figure 4b (a detailed discussion on the quantification of circulation is provided in Text S4). A circulation range of 1.73 × 10^3^ mm^2^/s to 1.95 × 10^3^ mm^2^/s was measured for inclined cilia/Δψ = +π/4, with a mean value of 1.85 × 10^3^ mm^2^/s calculated over 60 s. In comparison, an average circulation of only 1.6 × 10^3^ mm^2^/s was obtained for aligned cilia/Δψ = 0, which had previously demonstrated the highest mixing performance within that configuration (aligned cilia, Figure 3a). The enhanced mixing performance observed for the inclined cilia array under antiplectic actuation (Δψ = +π/4) can be attributed to the distinct flow behavior generated by this configuration, as evidenced by Section S2 and Figure S4, bottom panel. Unlike the aligned array, where the vortex formed further from the cilia tips and was often spatially broader, the inclined-antiplectic configuration produced a compact, high-vorticity vortex positioned directly above the cilia tips. Additionally, the velocity vectors were long, closely packed, and formed a well-defined swirl, indicating the improved rotational flow. These flow features were likely to promote local mixing and contribute to the improved mixing efficiency measured for this configuration. In summary, it can be suggested that metachronal actuation alone (as implemented in aligned cilia) was insufficient to generate the necessary fluid shear and vortex structures required for achieving improved mixing efficiency in the artificial cilia-based fluid mixing system. Instead, the integration of metachronal and temporal asymmetries with orientational asymmetry (as introduced in inclined cilia array configuration) was deemed essential to maximize the mixing performance. Moreover, this outcome highlighted a fundamental distinction between propulsion- and mixing-based applications of artificial cilia. Though metachronal motion alone has been shown to significantly enhance net fluid displacement in propulsion systems,^10^ improved mixing was found to necessitate a more intricate interplay of geometric and temporal actuation parameters.

**Figure 4:**
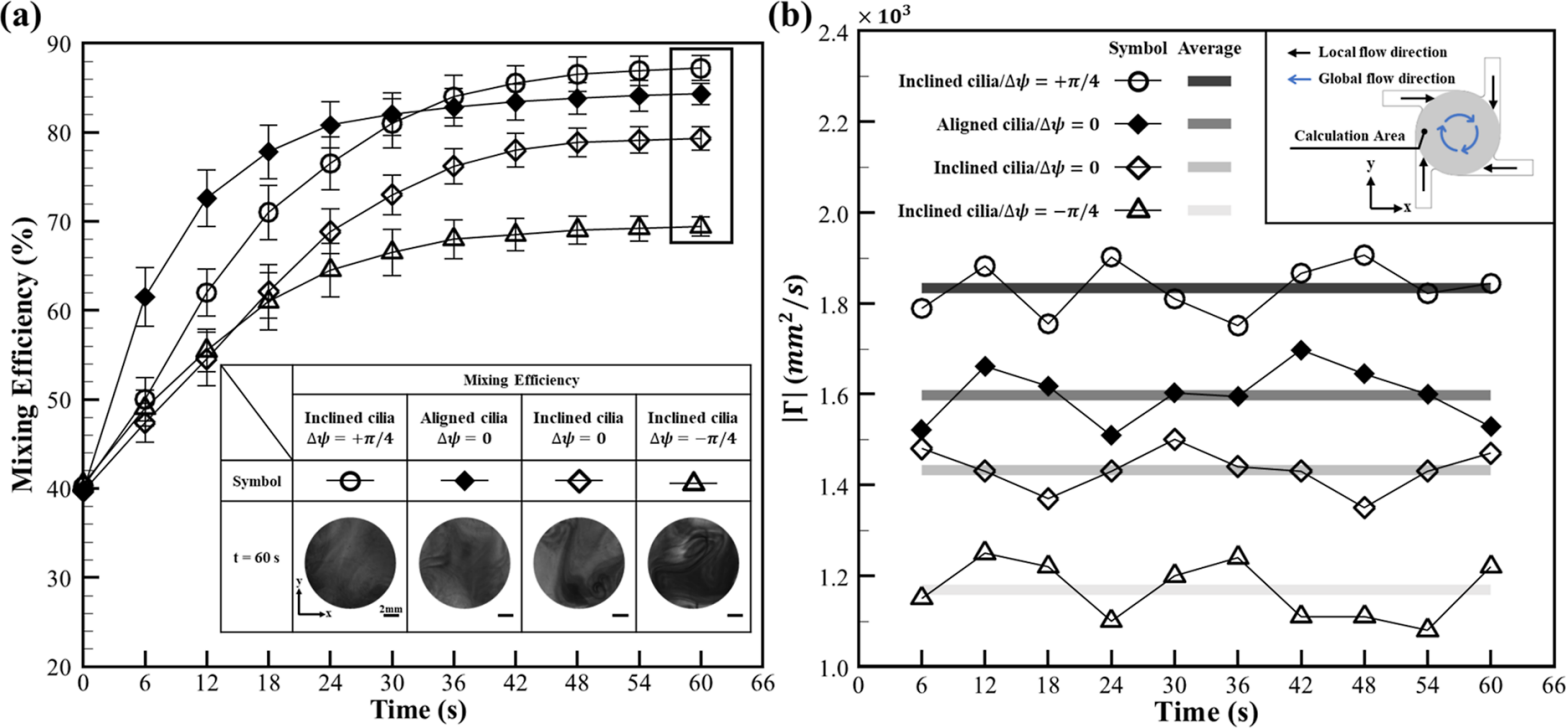
Analysis of mixing performance associated with inclined cilia array configuration and its respective hydrodynamic behavior. (a) The temporal evolution of mixing efficiency over 60 s for inclined cilia array configuration, actuated under mode Δψ = 0 and mode Δψ = +π/4, is illustrated. It was observed that after 60 s, inclined cilia/Δψ = +π/4 achieved a maximum mixing efficiency of 87%, whereas inclined cilia/Δψ = 0 reached 78%. Until 30 s, aligned cilia/Δψ = 0 was observed to exhibit superior performance compared to inclined cilia, however, beyond this point, inclined cilia/Δψ = +π/4 was observed to exhibit a modest improvement, ultimately surpassing the performance of aligned cilia. The error bar represents a unit standard deviation in both directions, calculated from the results of three independent experiments. Further, the representative experimental snapshots that were captured at 60 s are provided in the inset. The scale bar represents 5 mm. (b) Based on μPIV measurements, circulation values were computed over a circular region of interest (inset). A circulation range of 1.73 × 10^3^ mm^2^/s to 1.95 × 10^3^ mm^2^/s, with a mean of 1.85 × 10^3^ mm^2^/s, was measured for inclined cilia/Δψ = +π/4 actuation. In comparison, an average circulation of 1.6 × 10^3^ mm^2^/s was obtained for aligned cilia/Δψ = 0.

### The Magnetic Cilia-Assisted Photocatalytic Dye Degradation Performance

To functionally assess the mixing capability of the proposed artificial cilia platform beyond flow visualization metrics, a photocatalytic dye degradation experiment was conducted to evaluate the role of fluid agitation in enhancing mass transport and reaction kinetics. To demonstrate this experiment, MB was selected as a model organic pollutant, and Ti_3_C_2_ nanoparticles were utilized as the photocatalyst with visible light applied as the activation source. The objective of this experiment was not to evaluate the catalytic efficiency of the material itself but rather to demonstrate the influence of artificial ciliary motion on enhancing mass transport and mixing-driven surface reactions. Unlike the earlier mixing tests, which were primarily designed to emphasize tracer homogenization in the bulk fluid, this degradation assay was utilized to provide a functional perspective by assessing how effective mixing at the microscale could enhance real-time interactions at the catalyst–fluid interface. A detailed experimental procedure is provided at the end of this section and in Supporting Information Section S3, Figure S7. The experiment was conducted under two distinct environmental conditions, including a dark phase (a dark phase or condition was maintained (λ > 700 nm) by thoroughly wrapping the microfluidic device in multiple layers of aluminum foil and placing it inside a sealed, light-disabled chamber to prevent any exposure to ambient or stray light sources. This ensured that no photocatalytic activity occurred prior to illumination) followed by visible light exposure. Initially, all samples were maintained in darkness for 30 min to establish adsorption–desorption equilibrium between the Ti_3_C_2_ photocatalyst and the dye molecules. Following this equilibration, the visible light source embedded within the experimental setup was activated to initiate the photocatalytic reaction. Further, to quantify the extent of dye degradation under various cilia carpet configurations and actuation modes, the normalized concentration ratio, *C*/*C*_0_, was calculated, where *C*_0_ represents the initial concentration of MB and *C* denotes the concentration at a given time point. This parameter served as an indicator of the photocatalytic degradation efficiency and was used to assess the influence of artificial cilia-driven mixing across different experimental conditions, as illustrated in Figure 5a. A negligible degradation was observed in the experiments conducted in the dark across all actuation modes and cilia designs, with *C*/*C*_0_ values remaining above 0.9 at the end of 30 min. This result confirmed that any subsequent decrease in the dye concentration under illumination could be attributed solely to photocatalytic activity rather than passive adsorption. Upon exposure to visible light, substantial differences in the dye degradation performance were observed, depending on the structural design and actuation mode of the artificial cilia. As a control, the Ti_3_C_2_ material without any artificial cilia was tested under identical conditions. In this case, the normalized dye degradation concentration (*C*/*C*_0_) value was recorded to be 0.83 ± 0.02 after 120 min of illumination, corresponding to a degradation efficiency of 17%. Among the tested artificial cilia configurations, the combination of inclined cilia/Δψ = +π/4 was observed to demonstrate the highest degradation efficiency, with the *C*/*C*_0_ value reduced to 0.53 ± 0.01 (47% degradation efficiency). In contrast, the least dynamic actuation, such as mode Δψ = −π/4, applied to both array designs was observed to result in comparatively lower degradation, with final *C*/*C*_0_ values of 0.73 ± 0.02 (27% degradation efficiency) and 0.76 ± 0.01 (24% degradation efficiency), respectively. In quantitative terms, the degradation efficiency of inclined cilia/Δψ = +π/4 was approximately 2.7 times greater than that of the control group and 1.8 times greater than the least effective artificial cilia group (inclined cilia/Δψ = −π/4). To further assess the degradation mechanism (chemical reactivity) influenced by the hydrodynamics of artificial cilia, a detailed reaction kinetics analysis was performed. In particular, the temporal evolution of the dye concentration was systematically analyzed to quantify the rate at which the degradation process proceeded under various ciliary configurations. To facilitate this analysis, the experimental data were interpreted using a pseudo-first-order reaction model,^54–56^ a standard approach when the concentration of one reactant (in this case, the photocatalyst) remains effectively constant during the reaction. The rate constant “*k*” was subsequently determined (Section S4), which serves as a key kinetic parameter in characterizing the speed of the degradation process. This was accomplished by performing a linear regression analysis on the plot of (−ln­(*C*/*C*_0_)) versus time. This logarithmic transformation linearized the exponential decay behavior predicted by the pseudo-first-order model, enabling the slope of the resulting line to be directly interpreted as the apparent rate constant “*k*”. This approach was adopted in this study to serve as an additional quantitative metric for evaluating the degradation efficiency by determining the reaction rate constant under each experimental condition, as illustrated in Figure 5b. For the control case (where Ti_3_C_2_ alone was employed in the absence of artificial cilia), the rate constant was determined to be 0.0013 min^–1^, representing the lowest value compared to all of the tested artificial cilia configurations. Among the tested configurations, the combination of inclined cilia/Δψ = +π/4 was observed to exhibit the highest rate constant of 0.0046 min^–1^. Meanwhile, the next most effective configuration in the performance hierarchy, aligned cilia/Δψ = 0 (which was identified based on both mixing performance (Figure 4) and degradation efficiency (Figure 5a)) was observed to yield a slightly lower rate constant of 0.0042 min^–1^ (with a modest decrease of 8.7% compared to inclined cilia/Δψ = +π/4). On the other hand, configurations incorporating the least dynamic actuation, namely, mode Δψ = −π/I applied to both array designs, were observed to result in relatively reduced degradation rates, with rate constants of 0.0022 min^–1^ and 0.0016 min^–1^, respectively. Notably, these findings are consistent with those presented in Figure 5a. In quantitative terms, the rate constant achieved with inclined cilia/Δψ = +π/4 was found to be approximately 3.5 times greater than that of the control condition and 2.9 times higher than that of the least effective artificial cilia configuration (inclined cilia/Δψ = −π/4). These findings clearly highlight the synergistic advantage of the proposed magnetic cilia configurations in enhancing flow manipulation and reaction efficiency. As previously discussed in the context of mixing experiments, the influence of metachronal actuation alone was found to be limited in the absence of orientational asymmetry (aligned cilia with modes Δψ = +π/4 and Δψ = −π/4), a trend that was likewise evident in the dye degradation performance. In such cases, as seen in aligned cilia, the antiplectic and symplectic beatings did not yield substantial mixing improvement (Figure 3), which was also reflected in the degradation performance (Figure 5), where its effect was outperformed by synchronous beating.

**Figure 5:**
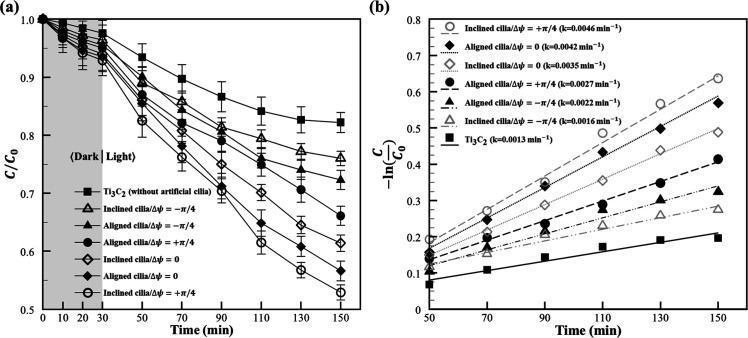
The photocatalytic dye degradation performance under different ciliary configurations and actuation modes. (a) The temporal evolution of the normalized MB dye concentration (*C*/*C*_0_) is depicted over 150 min under various artificial cilia configurations and actuation modes, as well as for a control group (Ti_3_C_2_ without artificial cilia). The combination of inclined cilia/Δψ = +π/4 was observed to demonstrate the highest degradation efficiency, with the C/C_0_ value reduced to 0.53 ± 0.01 after 120 min of illumination (47% degradation). In contrast, the least dynamic actuation (mode Δψ = −π/4) applied to both designs resulted in comparatively lower degradation. The control group exhibited the lowest degradation efficiency (*C*/*C*_0_ = 0.83 ± 0.02 after 120 min, 17% degradation). The error bar represents a unit standard deviation in both directions, calculated from the results of three independent experiments. (b) The temporal evolution of (−ln­(*C*/*C*_0_)) is plotted against time to analyze the reaction kinetics based on a pseudo-first-order model. Linear regression analysis was performed to determine the apparent rate constant “*k*” for each experimental condition. The lowest rate constant (0.0013 min^–1^) was determined for the control case. The highest rate constant (0.0046 min^–1^) was exhibited by inclined cilia/Δψ = +π/4, which was approximately 3.5 times greater than the control. Aligned cilia/Δψ = 0 yielded a slightly lower rate constant (0.0042 min^–1^). The least dynamic actuation (mode Δψ = −π/4) applied to both designs resulted in relatively reduced degradation rates. These kinetic findings were consistent with the observed degradation efficiencies in (a), highlighting the significant enhancement in the reaction rate achieved by the idealized cilia configuration and actuation mode.

### The Details of the Photocatalytic Experiment

The photocatalytic performance of Ti_3_C_2_ nanoparticles was evaluated by monitoring the degradation of MB under visible light illumination. MB was selected as the model organic contaminant for this study. A dilute solution of MB was prepared at a concentration of 40 μmol/L with a total volume of 3 mL and was introduced into the microfluidic device. To establish a baseline for absorbance, 0.035 mL of the prepared MB solution was withdrawn from the device and subjected to a second dilution by adding 0.315 mL of deionized water to a quartz cuvette. The diluted sample was then analyzed by using an UV–visible spectrophotometer (HITACHI U-3900H) to record the initial absorbance. The MB concentration was then determined by employing the Beer–Lambert law.^57^ Subsequently, 5 mg of the Ti_3_C_2_ photocatalyst was introduced into the microfluidic device, and the system was maintained under dark conditions for 30 min to allow for adsorption equilibrium. At regular intervals during the photocatalytic reaction, 0.035 mL aliquots of the MB solution was extracted, diluted with 0.315 mL of deionized water, and analyzed for absorbance using the spectrophotometer.

## Conclusion

In summary, a new magnetic cilia actuation framework was demonstrated for enhancing fluid manipulation in microfluidic systems. In particular, orientational asymmetry was embedded within the inclined cilia array design and combined with metachronal actuation modes to improve the mixing capability. To realize this operation, two different cilia array configurations, including aligned and inclined, were fabricated and actuated under three preprogrammed beating modes, such as synchronous (Δψ = 0), antiepileptic (Δψ = +π/4), and symplectic (Δψ = −π/4). Through comparative analysis, the mixing efficiency of inclined cilia/Δψ = +π/4 was observed to be improved, with the value reaching 87%. Further, an intensified vortical structure characterized by elevated circulation and shear stress was generated by this configuration. The improved fluid manipulation was further supported by photocatalytic dye degradation experiments, where a 3.5-fold increase in the degradation rate was observed compared to the control condition (Ti_3_C_2_ without cilia actuation) and a 1.38-fold improvement over the same mode (Δψ = +π/4) under aligned cilia array configuration. These outcomes underscore the importance of integrating temporal and orientational asymmetries (please refer to Figure S5 for mixing results under temporally symmetric actuation) to achieve improved mixing capabilities, offering a promising direction for multifunctional microfluidic applications.

## Supplementary Material





## Notes

Beating and mixing performance of the magnetic cilia array under different actuation modes and spatial configurations (MP4)
Analytical estimation of magnetic field distribution during magnetization; schematic representation of the magnetic field direction generated by two axially magnetized cylindrical permanent magnets during the magnetization process; schematic of the magnetization setup for artificial cilia arrays; schematic illustration of the cilia array beating in three different modes; ensemble-averaged velocity vector fields and vorticity contours generated by a single artificial cilia array across both designs and three metachronal beating modes; comparison of mixing efficiencies at 60 s for the two best-performing artificial cilia array configurations under conditions of temporal asymmetry and temporal symmetry; spatially averaged angular velocity (Ω_z_) measured at 60 s for different artificial cilia array configurations; quantification of dye concentration based on UV–visible absorbance measurements; temporal evolution of absorbance spectra for the inclined cilia array actuated with a phase offset of Δψ = +π/4; comparative statistical analysis between the two best-performing configurations, namely, the inclined cilia with phase offset Δψ = +π/4 and the aligned cilia with Δψ = 0; time-lapse velocity vector plots and spatially averaged velocity magnitudes for the aligned cilia array configurations; photocatalytic dye degradation reaction kinetics analysis; comparison of fluid velocity magnitudes measured in the present study with values reported in the literature; and comparison of mixing efficiencies reported in previous studies employing magnetically actuated artificial cilia (PDF)
