# Supplementary material for: Magnetic Cilia with Programmable Beating Patterns for Vortex-Driven Mixing in Microfluidics
Source: Langmuir. 2025 Jul 31;41(32):21562–75. doi: 10.1021/acs.langmuir.5c02350 (PMC12369015; doi:10.1021/acs.langmuir.5c02350)
Supplement: Supplementary file 2 [file la5c02350_si_002.pdf]

Supporting Information

Magnetic Cilia with Programmable Beating Patterns for Vortex-Driven Mixing in Microfluidics

Dineshkumar Loganathan, Tung Ou Yang, Chia-Yun Chen, and Chia-Yuan Chen**

Number of pages: **17**
Number of figures: **09**
Number of tables: **02**

Table of Contents

Section S1. Analytical Estimation of Magnetic Field Distribution During Magnetization

Figure S1. Schematic representation of the magnetic field direction generated by two axially magnetized cylindrical permanent magnets during the magnetization process

Figure S2. Schematic of the Magnetization Setup for Artificial Cilia Arrays

Figure S3. Schematic illustration of the cilia array beating in three different modes

Section S2. Ensemble-averaged velocity vector fields and vorticity contours generated by a single artificial cilia array across both designs and three metachronal beating modes

Figure S4. Ensemble-averaged velocity vector fields and vorticity contours generated by a single artificial cilia array across both designs and three metachronal beating modes

Figure S5. Comparison of mixing efficiencies at 60 seconds for the two best-performing artificial cilia array configurations under conditions of temporal asymmetry and temporal symmetry

Figure S6. Spatially averaged angular velocity (Ω_z) measured at 60 seconds for different artificial cilia array configurations

Section S3. Quantification of Dye Concentration Based on UV–Visible Absorbance Measurements

Figure S7. Temporal evolution of absorbance spectra for the inclined cilia array actuated with a phase offset of $\Delta\psi = +\pi/4$

Figure S8. A comparative statistical analysis between the two best-performing configurations, namely, the inclined cilia with phase offset $\Delta\psi = +\pi/4$ and the aligned cilia with $\Delta\psi = 0$

Figure S9. Time-lapse velocity vector plots and spatially averaged velocity magnitudes for the aligned cilia array configurations

Section S4. Photocatalytic dye degradation reaction kinetics analysis

Table S1. Comparison of fluid velocity magnitudes measured in the presented study with values reported in the literature

Table S2. Comparison of mixing efficiencies reported in previous studies employing magnetically actuated artificial cilia

Section S1. Analytical Estimation of Magnetic Field Distribution During Magnetization

To gain insight into the magnetic field distribution experienced by the artificial cilia arrays during the magnetization process, an analytical model was employed to estimate the axial magnetic field generated by a pair of axially magnetized cylindrical permanent magnets. The cilia arrays were magnetized by employing two neodymium–iron–boron (NdFeB) permanent magnets, each having a diameter of 25 mm and a length of 75 mm. These magnets were positioned in an opposing face-to-face configuration, separated by a 15 mm gap, with the cilia array placed at the midpoint along the central axis between them. The magnetic field B(z) along the axial direction (z-axis) between the two magnets can be approximated using the following expression

$$B(z) = \frac{\mu_0 \times M}{2}\left[\frac{z + L/2}{\sqrt{(z + L/2)^2 + R^2}} - \frac{z - L/2}{\sqrt{(z - L/2)^2 + R^2}}\right] \tag{S1}$$

where μ is the vacuum permeability, M is the magnetization of the permanent magnet, R is the radius of the magnet, L is the length of the magnet, and z is the distance along the central axis. This expression (S1) represents the axial component of the magnetic field generated by a single magnet and can be adapted to approximate the total field resulting from two magnets arranged symmetrically about z=0. This equation provides an estimation of the magnetic field magnitude experienced by the cilia array during the magnetization step. Further, a schematic illustration of the field configuration corresponding to this model is shown in Supplementary Figure S1.

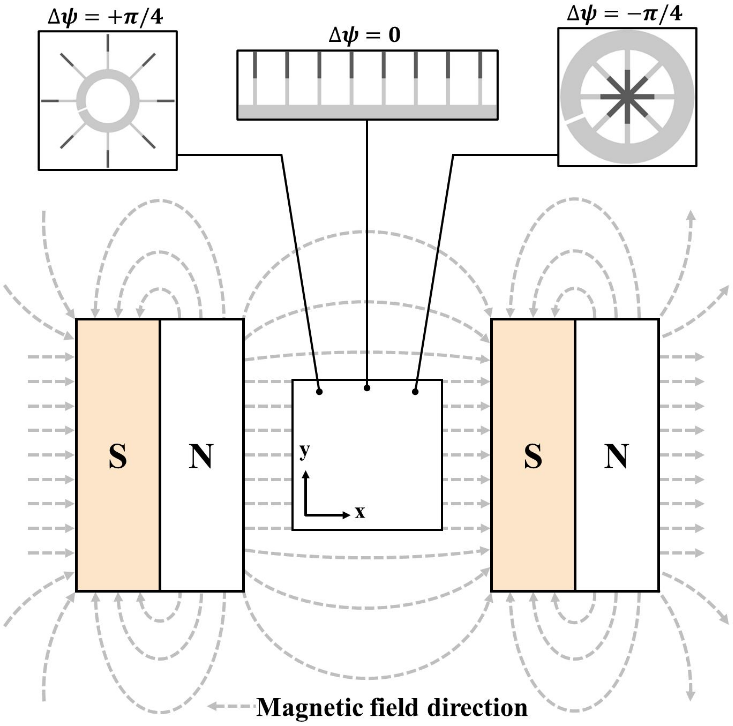

Figure S1: Schematic representation of the magnetic field direction generated by two axially magnetized cylindrical permanent magnets during the magnetization process. The grey dashed lines with arrows indicate the direction of the magnetic field vectors. Insets illustrate the resulting magnetization patterns of the artificial cilia arrays for different modes ($\Delta\psi=+\pi/4$, $\Delta\psi=0$, and $\Delta\psi=-\pi/4$), demonstrating how the magnetic field was aligned with the cilia for effective magnetization.

Figure S2. Schematic of the Magnetization Setup for Artificial Cilia Arrays.

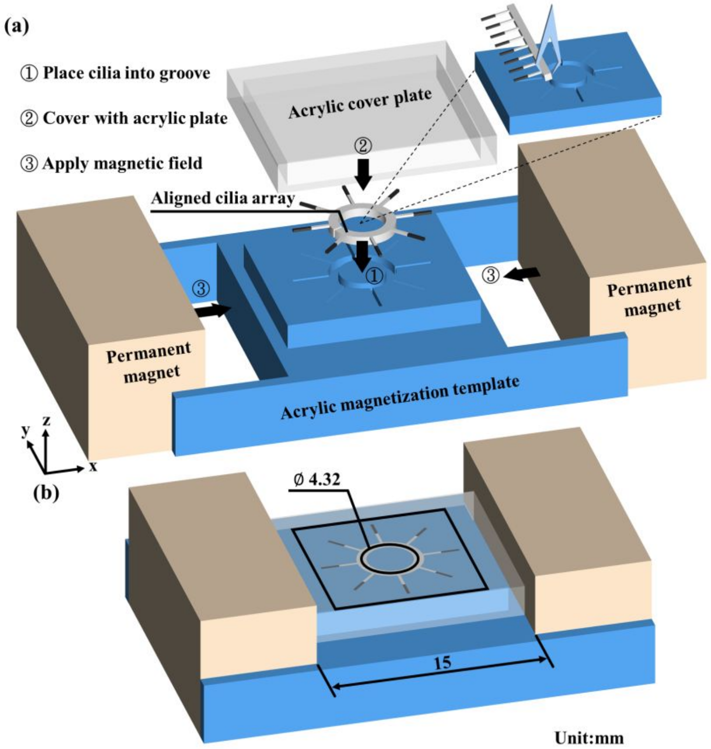

Figure S2. Schematic of the Magnetization Setup for Artificial Cilia Arrays. As illustrated, the artificial cilia array was placed into a circular groove fabricated on an acrylic magnetization template. This groove served to hold the base of the cilia array in position during the magnetization process. To further secure the array and minimize movement or deformation, an acrylic cover plate was positioned on top of the setup, applying a gentle downward force. The diameter of the circular groove, which defines the constrained region for magnetization, was 4.32 mm, as shown in (ii). Both the template and the cover plate were made of acrylic due to its non-magnetic properties and ease of fabrication. This arrangement allowed the cilia array to remain properly aligned during exposure to the magnetic field generated by the permanent magnets.

Figure S3. **Schematic illustration of the cilia array beating in three different modes**

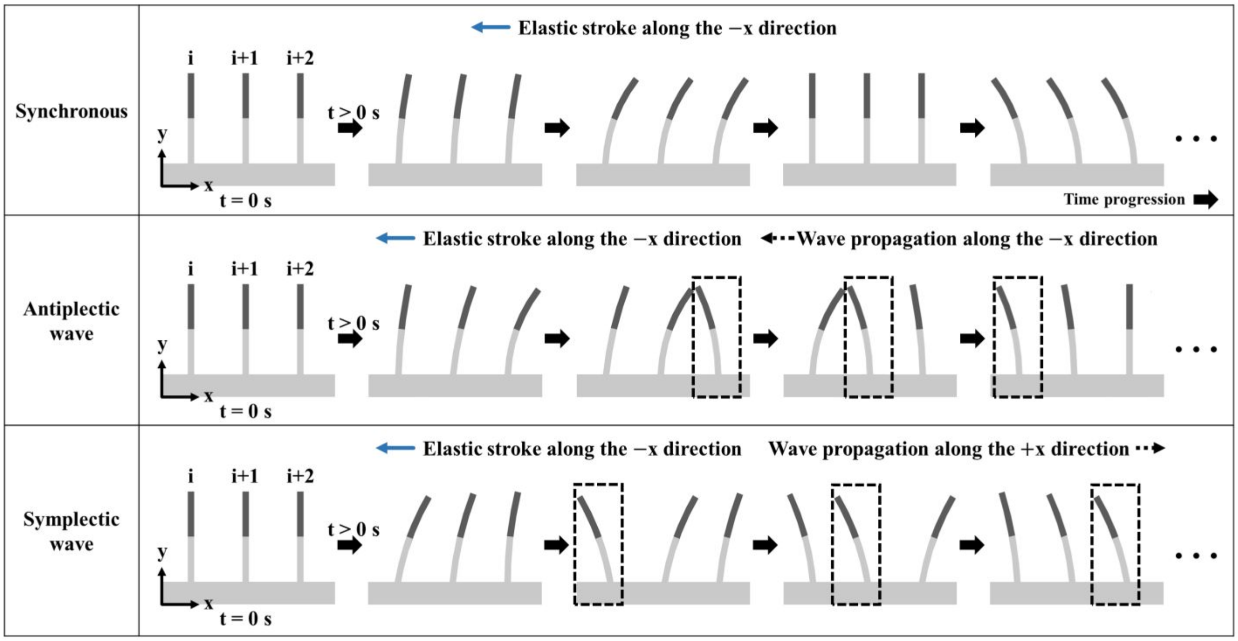

Figure S3: Schematic illustration of the phase relationships between neighboring cilia and the direction of wave propagation relative to the elastic (recovery stroke) for different metachronal modes. The top panel shows an aligned cilia array with individual cilia labeled (i, i+1, i+2) for synchronous beating. The middle panel depicts the antiplectic metachronal wave, where the wave of motion propagates in the same direction as the elastic stroke (along the -x direction). The bottom panel illustrates the symplectic metachronal wave, where the wave of motion propagates in the direction opposite to the elastic stroke (along the +x direction). This visual aid assists in distinguishing between the two metachronal modes and conveys their underlying kinematic differences.

Section S2. Ensemble-averaged velocity vector fields and vorticity contours generated by a single artificial cilia array across both designs and three metachronal beating modes. To better understand the fluidic behavior generated by each artificial cilia configuration, flow field experiments were conducted using a single cilia array placed inside the microfluidic chamber (Supplementary Figure S4). Unlike the combined array arrangement employed for evaluating mixing and photocatalytic dye degradation performance, this set of experiments focused solely on resolving the flow structures generated by individual array configurations. These studies aimed to assess the vorticity distribution and velocity vector fields induced by each actuation mode, thereby providing additional insight into the fluid manipulation characteristics of the tested designs. Velocity vectors overlaid with vorticity contours were obtained for all six configurations, including aligned and inclined cilia arrays actuated synchronously ($\Delta\psi = 0$), antiplectically ($\Delta\psi = +\pi/4$), and symplectically ($\Delta\psi = -\pi/4$). A representative reference velocity of 12 mm/s was included for scale, and the color map ranged from −8 to +8 s^{-1} to visualize the spatial distribution of vorticity. All results represent ensemble-averaged fields obtained over 18 beating cycles. In the **aligned synchronous configuration ($\Delta\psi = 0$)**, a pronounced vortex structure was observed in the upper central region of the field of view. This vortex was relatively circular in shape and concentrated within a localized area spanning 5–7 mm in diameter. The vorticity value at its core can be observed to reach -6 to –8 s^{-1} (the negative sign indicates the direction of the vortex). Further, the velocity vectors within the vortex were dense and tangentially aligned, forming a swirling pattern consistent with rotational motion. This observed pattern is in line with the previously measured mixing efficiency of 84% for this configuration. For the **aligned antiplectic mode ($\Delta\psi = +\pi/4$)**, the main vortex was observed to be elongated along the horizontal axis. The vorticity magnitude in this configuration was moderately lower, with peak values ranging from -1 to -4 s^{-1} (the negative sign indicates the direction of the vortex). Although rotational motion was still present, the flow exhibited greater directional extension, which reduced its capacity to fold and recirculate fluid efficiently. This behavior corresponds with its measured mixing efficiency of 75%. In the **aligned symplectic mode ($\Delta\psi = -\pi/4$)**, a similarly elongated vortex can be seen with more diffuse and lower values ranging from -1 to 2 s^{-1}. Compared to the other aligned configurations, this mode produced a more translational flow with reduced stirring strength, which is consistent with its lower mixing efficiency of 71%. For the **inclined synchronous configuration ($\Delta\psi = 0$)**, a localized vortex appeared near the cilia tips at the bottom of the channel. The vortex occupied a confined region of approximately 4–5 mm in diameter, characterized by strong positive vorticity in the range of 6–8 s^{-1}. The surrounding velocity vectors were tightly curved and well-aligned, forming a compact flow structure close to the channel boundary. Here, the term 'compact vortex' refers to a rotational flow confined within a limited spatial area. This localized stirring is beneficial for entraining fluid from the surface and enhancing mixing near the boundary, and corresponds with the measured mixing efficiency of 78%. The **inclined antiplectic**

configuration (Δψ = +π/4) generated the strongest flow features among all tested modes. A dense and tightly coiled vortex was observed directly above the cilia tips, with vorticity magnitudes exceeding 8 s^{-1} at the core. The velocity vectors were long, closely packed, and formed a well-defined swirl, indicating the improved rotational flow. This high local vorticity and strongly circular vector alignment are indicative of better stirring in the near-cilia region. This behavior aligns with the highest mixing efficiency recorded among all modes, measured at 87%. In contrast, the **inclined symplectic configuration (Δψ = −π/4)** produced an elongated vortex extending from the cilia tips. The vorticity was comparatively lower, ranging from -2 to 1 s^{-1}, and the velocity vectors exhibited a more stretched pattern, suggesting horizontal transport of fluid rather than confined swirling. This flow type is more representative of directional sweeping than localized stirring and corresponds with the lowest mixing efficiency (70%) among the inclined cilia cases. In summary, the induced flow fields varied noticeably across the tested configurations, with synchronous modes and the inclined antiplectic mode producing more compact, high-magnitude vortices that supported improved mixing behavior. In contrast, the symplectic modes exhibited weaker rotational strength and were more suited for directional fluid transport. These findings support and complement the dye-based mixing measurements and provide a detailed flow-based interpretation of the distinct fluidic behaviors induced by artificial cilia arrays.

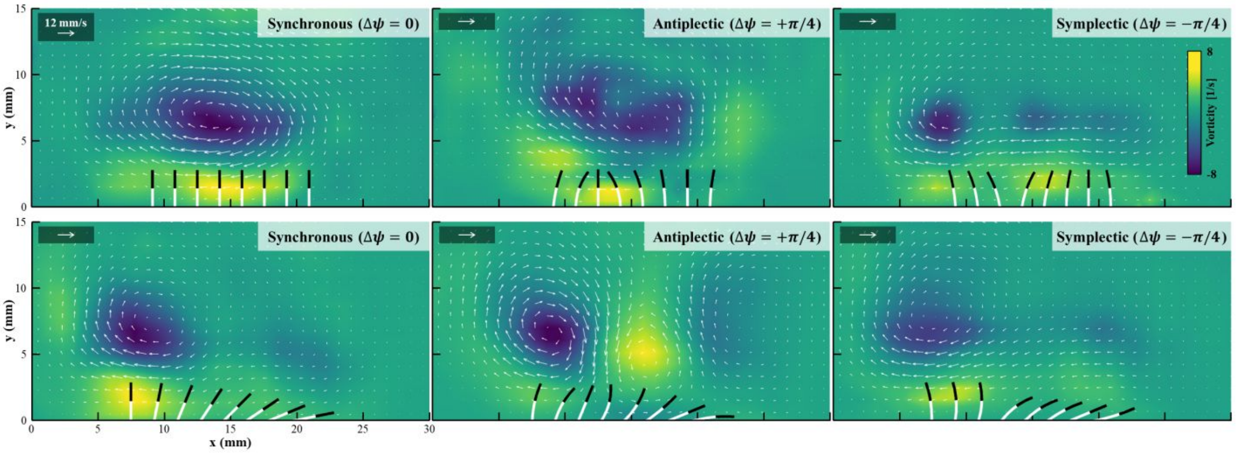

Figure S4. Ensemble-averaged velocity vector fields and vorticity contours generated by a single artificial cilia array across both designs and three metachronal beating modes. The top and bottom rows correspond to aligned and inclined cilia arrays, respectively, each actuated under synchronous (Δψ = 0), antiplectic (Δψ = +π/4), and symplectic (Δψ = −π/4) modes. The velocity vectors illustrate the spatial flow direction and relative speed, with a reference vector of 12 mm/s provided for scale. Vorticity distributions (color bar, −8 to +8 s^{-1}) highlight the strength and polarity of induced rotational flow. Compact, high-vorticity vortices were observed for the aligned and inclined synchronous modes as well as the inclined antiplectic configuration, correlating with their improved mixing performances. In contrast,

elongated and lower-vorticity structures were generated in the antiplectic and symplectic modes of the aligned array and the symplectic mode of the inclined array, suggesting reduced mixing and potential directional flow. All flow fields were ensemble-averaged over 18 beating cycles.

Figure S5. Comparison of mixing efficiencies at 60 seconds for the two best-performing artificial cilia array configurations under conditions of temporal asymmetry and temporal symmetry

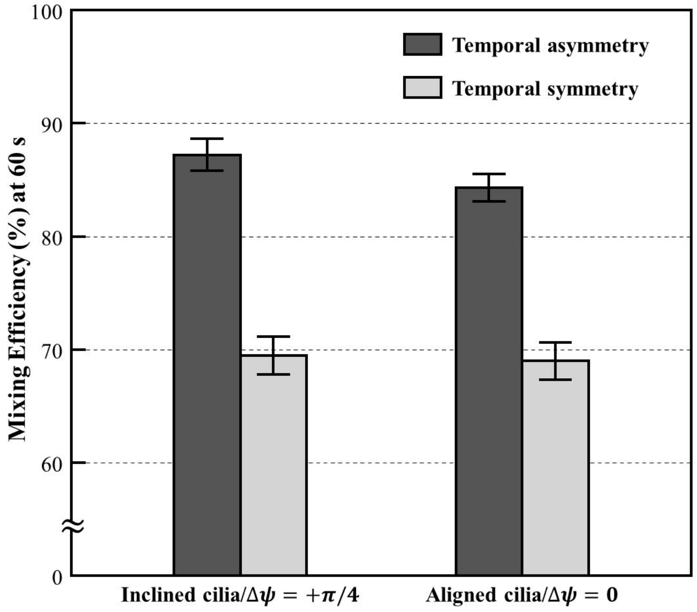

Figure S5: Comparison of mixing efficiencies at 60 seconds for the two best-performing artificial cilia array configurations under conditions of temporal asymmetry and temporal symmetry. For the inclined cilia with $\Delta\psi=+\pi/4$, the mixing efficiency was measured to be 87.3 ± 0.5% under temporal asymmetry, significantly higher than the 69.6 ± 0.8% observed under temporal symmetry. Similarly, for the aligned cilia with $\Delta\psi=0$, the mixing efficiency was 84.8 ± 0.4% with temporal asymmetry, compared to 68.0 ± 0.7% with temporal symmetry. This figure demonstrates the critical role of temporal asymmetry in enhancing the mixing performance of the artificial cilia arrays. Error bars represent the standard deviation of three independent measurements.

Figure S6. Spatially averaged angular velocity (Ω_z) measured at 60 seconds for different artificial cilia array configurations

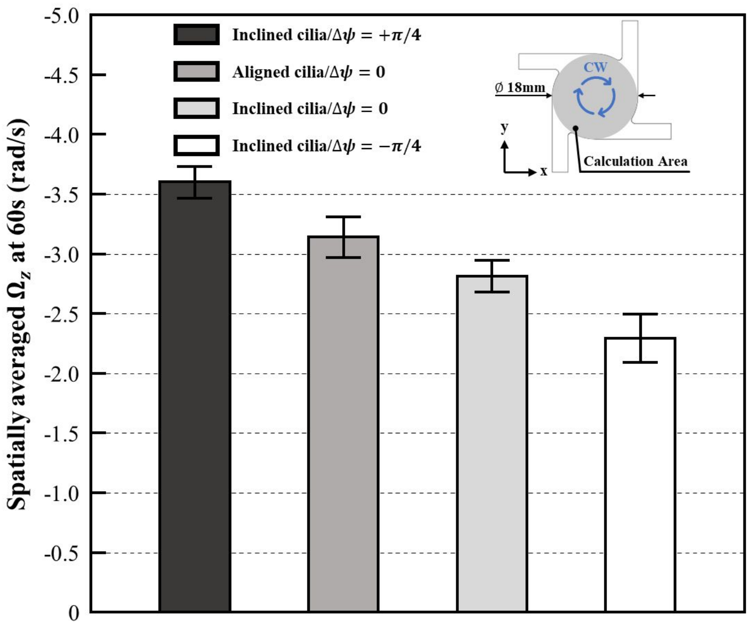

Figure S6. Spatially averaged angular velocity (Ω_z) measured at 60 seconds for different artificial cilia array configurations. This metric was computed from locally resolved micro-PIV velocity fields to complement the net circulation values by providing a spatially distributed measure of rotational strength within the fluid domain. Notably, the inclined cilia array actuated with a phase offset of $\Delta\psi = +\pi/4$ exhibited the highest angular velocity, recorded as -3.6 ± 0.05 rad/s, indicating the formation of a strong counterclockwise (CCW) vortex. The inset illustrates the calculation region (Ø 18 mm) within the microfluidic chamber, along with the coordinate system and the direction of vortex rotation. Error bars represent the standard deviation from three repeated measurements.

Section S3. Quantification of Dye Concentration Based on UV–Visible Absorbance Measurements

To quantify the degradation of methylene blue (MB) dye during the photocatalytic experiments, dye concentration at different time points was determined based on the Beer–Lambert law, which establishes a direct linear relationship between absorbance and solute concentration. Accordingly, the relative dye concentration was calculated by employing the following expression

$$C/C_0=A/A_0 \tag{S2}$$

where C_0 and A_0 represent the initial concentration and absorbance of the MB dye solution, respectively, and C and A correspond to the concentration and absorbance at a later time. This ratio was employed to assess the extent of photocatalytic degradation over time. At the beginning of the experiment, a 40 μM MB solution with a total volume of 3 mL was introduced into the microfluidic system. At designated time points, 0.035 mL of the reaction solution was extracted from the mixing chamber and transferred into a white micro-quartz colorimetric tube. To ensure sufficient optical volume and consistent path length, 0.315 mL of deionized water was added, bringing the total sample volume to 0.35 mL. This diluted sample was then placed in a UV–visible spectrophotometer (Model U-3900, Hitachi High-Technologies Corp., Japan), and the absorbance was measured at 663.5 nm, which corresponds to the maximum absorbance (λ_{max}) of MB dye. This wavelength provided high sensitivity for detecting small changes in dye concentration. A typical example of this measurement is presented in **Figure S7**, which shows the absorbance spectra recorded over time for the inclined cilia array actuated with a phase offset of $\Delta\psi = +\pi/4$. The figure demonstrates a progressive decrease in absorbance at the peak wavelength (~660 nm), dropping from 0.11 a.u. at t = 0 min to 0.05 a.u. at t = 150 min. These spectra clearly illustrate the temporal reduction in dye concentration. The corresponding figure insets further corroborate this observation by showing visual fading of the dye within the microfluidic channel. All absorbance measurements were performed in triplicate, and the average value was employed for concentration analysis. This approach enabled accurate tracking of the temporal evolution of MB concentration, thereby quantifying the effectiveness of photocatalytic degradation under various cilia actuation conditions.

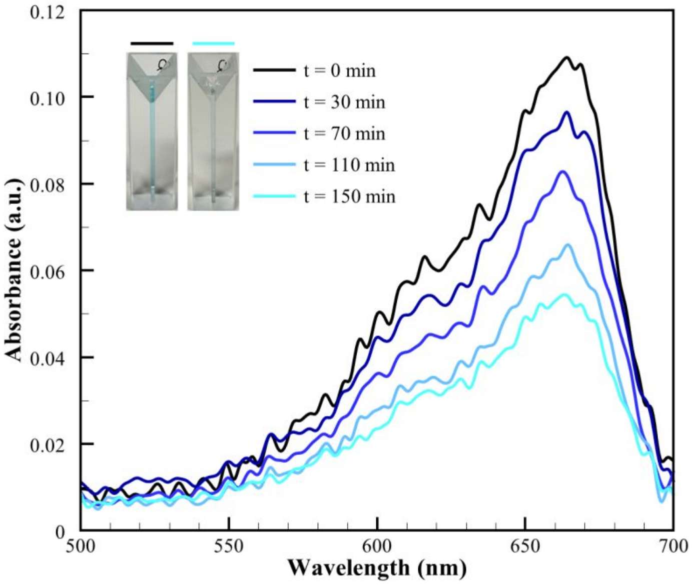

Figure S7. Temporal evolution of absorbance spectra for the inclined cilia array actuated with a phase offset of $\Delta\psi = +\pi/4$. A progressive decrease in absorbance at the peak wavelength (~660 nm) was observed, indicating a reduction in methylene blue concentration due to photocatalytic degradation. The absorbance decreases from 0.11 a.u. at t = 0 min (black line) to 0.05 a.u. at t = 150 min (light blue line). The legend denotes the time points (0, 30, 70, 110, and 150 min) corresponding to each spectrum. The inset displays photographic images of the microfluidic channel at selected time points, visually confirming the decline in dye concentration.

Figure S8. A comparative statistical analysis was performed between the two best-performing configurations, namely, the inclined cilia with phase offset Δψ = +π/4 and the aligned cilia with Δψ = 0, to validate the observed improvements in both mixing efficiency and photocatalytic dye degradation.

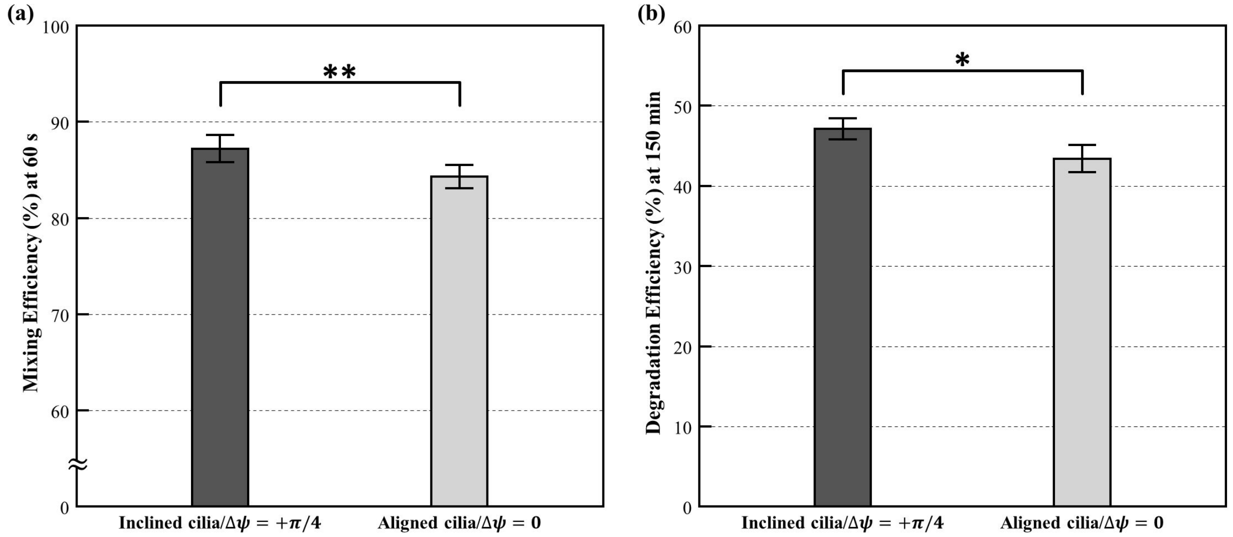

Figure S8. A comparative statistical analysis was performed between the two best-performing configurations, namely, the inclined cilia with phase offset Δψ = +π/4 and the aligned cilia with Δψ = 0, to validate the observed improvements in both mixing efficiency and photocatalytic dye degradation. As shown in this figure, the mixing efficiency measured at 60 seconds for inclined cilia/Δψ=+π/4 was found to be 87.3 ± 0.5%, while aligned cilia/Δψ=0 yielded 84.8 ± 0.4%, with a statistical difference of ($p \leq 0.01$, denoted by **). Similarly, in the photocatalytic degradation experiments, the relative concentration (C/C_0) at 150 minutes was 0.53 ± 0.01 (and the corresponding degradation efficiency was 47%) for inclined cilia/Δψ=+π/4 and 0.59 ± 0.03 (the corresponding degradation efficiency was 41%) for aligned cilia/Δψ=0, also indicating a statistical difference of ($0.01 < p \leq 0.05$, denoted by *).

Figure S9. **Time-lapse velocity vector plots and spatially averaged velocity magnitudes for the aligned cilia array configurations**

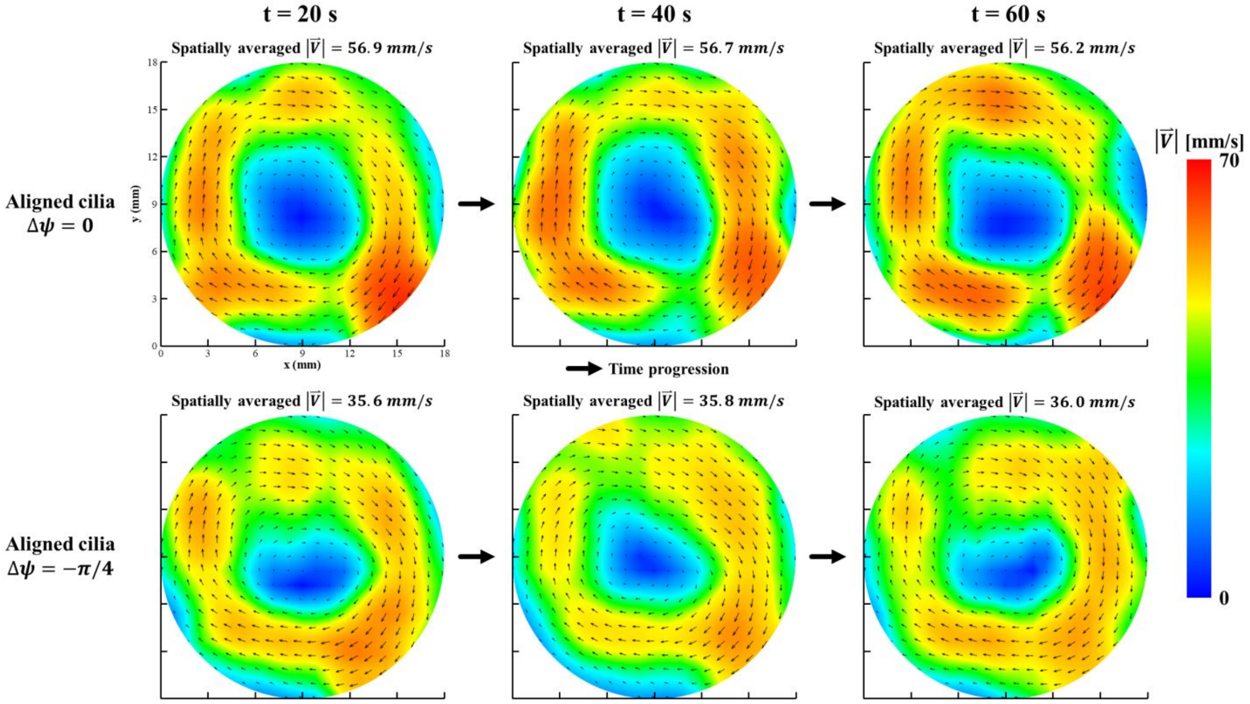

Figure S9. **Time-lapse velocity vector plots and spatially averaged velocity magnitudes for the aligned cilia array configurations.** The top row shows the flow field evolution for the Δψ = 0 configuration at t = 20 s, 40 s, and 60 s. The bottom row shows corresponding plots for Δψ = –π/4. Color maps represent the magnitude of velocity vectors (mm/s). The Δψ = 0 configuration demonstrated consistently high average velocities (56.9 mm/s at t = 20 s, 56.7 mm/s at t = 40 s, and 56.2 mm/s at t = 60 s), while the Δψ = –π/4 configuration generated significantly lower values (35.6 mm/s, 35.8 mm/s, and 36.0 mm/s at the respective time points), correlating with weaker flow and reduced mixing.

Section S4. **Photocatalytic dye degradation reaction kinetics analysis.** To evaluate the degradation mechanism influenced by artificial cilia-induced hydrodynamics, a reaction kinetics analysis was conducted. Specifically, the temporal evolution of dye concentration was assessed by employing a pseudo-first-order kinetic model, which is appropriate when the catalyst concentration remains effectively constant throughout the reaction. Under this assumption, the rate of degradation depends solely on the dye concentration and follows the form shown below

$$-dC/dt = kC \tag{S3}$$

Upon integration,

$$-\ln(C/C_0) = kt \tag{S4}$$

where C is the dye concentration at time t, C_0 is the initial concentration, and k is the apparent rate constant. This linearized relationship enabled a straightforward determination of k through linear regression of the experimental data plotted as $-\ln(C/C_0)$ versus time. The dashed lines in Figure 5b (from the main manuscript) correspond to these regression fits for each tested configuration and represent the best-fit trends employed to extract the respective k values.

Table S1. Comparison of fluid velocity magnitudes measured in the presented study with values reported in the literature for artificial cilia systems with similar dimensions and actuation conditions. The measured 'fluid velocity' and 'scaled fluid velocity' in the presented study were found to be in a similar range to those reported in prior experimental studies, thereby supporting the reliability of the flow measurements.

**Cilia Actuation Method**	**Fluid velocity (V)**	**Cilia Length (L)**	**Scaled Fluid Velocity V_{scaled}=(V/L)**	**Reference**
Magnetic	Up to 3000 μm/s	350 μm	8.57 1/s	(S1)
Magnetic	Up to 1400 μm/s	100 μm	14 1/s	(S2)
Electric	0.33 ~ 1.94 mm/s	1.5 mm	0.22 ~ 1.29 1/s	(S3)
Magnetic	0.95 mm/s	1 mm	0.95 1/s	(S4)
Magnetic	0.13 mm/s ~ 11.02 mm/s	2.8 mm	0.046 ~ 3.93 1/s	This work

Table S2. Comparison of mixing efficiencies reported in previous studies employing magnetically actuated artificial cilia with different actuation modes, cilia dimensions, and array densities, benchmarked against the present work

**Artificial cilia actuation method**	**Length of cilia**	**Number of cilia employed**	**Beating pattern**	**Mixing efficiency**	**Reference**
Magnetic Actuation	300 μm	36	Metachronal beating	~75%	(S5)
Magnetic Actuation	20 ~ 40 μm	more than 1000 cilia	Static Structural Response Mode	~87%	(S6)
Magnetic Actuation	1.5mm	8	Single repetitive beating (non-metachronal)	~85%	(S7)
Magnetic Actuation	2.8mm	32	Metachronal beating /Synchronous beating	~87%	This study

References

S1. Zhang, S.; Cui, Z.; Wang, Y.; den Toonder, J. M. J., Metachronal actuation of microscopic magnetic artificial cilia generates strong microfluidic pumping. Lab on a Chip 2020, 20 (19), 3569-3581.

S2. Hanasoge, S.; Hesketh, P. J.; Alexeev, A., Microfluidic pumping using artificial magnetic cilia. Microsystems & Nanoengineering 2018, 4 (1), 11.

S3. Ren, Z.; Zhang, M.; Song, S.; Liu, Z.; Hong, C.; Wang, T.; Dong, X.; Hu, W.; Sitti, M., Soft-robotic ciliated epidermis for reconfigurable coordinated fluid manipulation. Science Advances 2022, 8 (34), eabq2345.

S4. Dong, X.; Lum, G. Z.; Hu, W.; Zhang, R.; Ren, Z.; Onck, P. R.; Sitti, M., Bioinspired cilia arrays with programmable nonreciprocal motion and metachronal coordination. Science Advances 2020, 6 (45), eabc9323.

S5. Wang, T.; Aggarwal, I.; Steur, E.; Homan, T.; Onck, P. R.; den Toonder, J. M. J.; Wang, Y., Designing enhanced mixing in stagnant microfluidic environments: an artificial cilia approach. Lab on a Chip 2025, DOI: 10.1039/d5lc00186b

S6. Chen, G.; Dai, Z.; Li, S.; Huang, Y.; Xu, Y.; She, J.; Zhou, B., Magnetically Responsive Film Decorated with Microcilia for Robust and Controllable Manipulation of Droplets. ACS Applied Materials

& Interfaces 2021, 13 (1), 1754-1765.
S7. Rahbar, M.; Shannon, L.; Gray, B. L., Microfluidic active mixers employing ultra-high aspect-ratio rare-earth magnetic nano-composite polymer artificial cilia. Journal of Micromechanics and Microengineering 2014, 24 (2), 025003.
